# Microbial metabolite–driven mechanisms linking the gut microbiome to atherosclerosis: multi-omic and translational perspectives

**DOI:** 10.1080/19490976.2026.2718621

**Published:** 2026-08-19

**Authors:** Mar Masiá, Félix Gutiérrez

**Affiliations:** a Hospital General Universitario de Elche and Universidad Miguel Hernández, Alicante, Spain; b CIBERINFEC, ISCIII, Madrid, Spain; c Fundación para el Fomento de la Investigación Sanitaria y Biomédica de la Comunitat Valenciana (FISABIO), Valencia, Spain

**Keywords:** Gut microbiome, atherosclerosis, microbial metabolites, metabolomics, immune activation, vascular inflammation, multi-omics, precision medicine

## Abstract

Atherosclerotic cardiovascular disease remains the leading cause of mortality worldwide, and a substantial residual risk persists despite optimal management of traditional risk factors. Increasing evidence implicates the gut microbiome as a mechanistic contributor to atherogenesis, not merely through taxonomic shifts but via the production of bioactive microbial metabolites that link diet, microbial metabolism, and host vascular biology. These metabolites have emerged as central effectors of the gut–artery axis, influencing intestinal barrier integrity, systemic immunity, lipid handling, and thrombosis. Among the best-characterized pathways, trimethylamine *N*-oxide and phenylacetylglutamine have been robustly linked to macrophage lipid accumulation, platelet hyperreactivity, and adverse cardiovascular outcomes. More recently, imidazole propionate, a histidine-derived microbial metabolite, has emerged as a candidate mediator of vascular inflammation and plaque development through imidazoline-1 receptor–dependent activation of mTORC1 signaling, supported by mechanistic and experimental evidence. Advances in metagenomics, metabolomics, and proteomics now enable systems-level interrogation of microbiome–host interactions, facilitating causal inference through integrative metabolite–protein and pathway-level analyses. These approaches have revealed reproducible molecular networks associated with subclinical and clinical atherosclerosis, providing a framework for biomarker discovery and therapeutic targeting. People with HIV represent a particularly informative human model, in which persistent gut barrier disruption and dysbiosis sustain immune activation and confer excess cardiovascular risk, with distinct microbial and metabolite signatures linked to vascular inflammation and plaque progression. This review synthesizes current evidence linking gut microbial function to atherosclerosis, with a specific focus on metabolite-driven mechanisms, multi-omic integration, and translational relevance. We highlight emerging biomarkers and therapeutic strategies targeting microbial metabolic pathways and discuss methodological challenges that must be addressed to advance the gut–artery axis toward precision cardiovascular medicine.

## 1. Introduction

Atherosclerotic cardiovascular disease (ASCVD) remains the leading cause of morbidity and mortality worldwide, despite substantial advances in prevention and treatment.^1-4^ Although established risk factors such as dyslipidemia, hypertension, smoking, and diabetes account for much of this burden, they do not fully explain inter-individual variability in disease susceptibility and progression. In this context, the gut microbiome has emerged as an important modulator of cardiometabolic health, influencing host metabolic, immune, and vascular pathways.^5-7^

Mechanistic evidence indicates that microbiome-associated effects on atherogenesis converge on three interconnected axes: disruption of intestinal barrier integrity with translocation of microbial products, activation of innate and adaptive immune responses, and production of bioactive microbial metabolites that directly affect vascular and metabolic function.^8-13^ Among these, microbial metabolites provide a particularly compelling mechanistic link between environmental exposures and host physiology. Compounds derived from dietary substrates—including trimethylamine *N*-oxide (TMAO), phenylacetylglutamine (PAGln), and imidazole propionate (ImP)—have been associated with endothelial dysfunction, inflammation, and adverse cardiovascular outcomes across experimental and human studies.^14-19^

However, several challenges complicate interpretation of these associations. Many of the implicated pathways—such as immune activation, metabolic inflammation, and altered microbial metabolism—are shared across cardiometabolic diseases, including obesity and metabolic dysfunction–associated steatotic liver disease, raising questions regarding specificity. In addition, substantial heterogeneity across studies—driven by differences in diet, geography, host factors, comorbidities, and analytical approaches—limits reproducibility, particularly at the taxonomic level.^9-11^ ^,^ ^13^ ^,^ ^20^ ^,^ ^21^ These considerations underscore the need to move beyond descriptive compositional analyses toward integrative, functionally oriented frameworks.

Diet represents a key upstream determinant of microbial metabolic capacity, governing substrate availability and thereby shaping the production of metabolites with downstream vascular effects.^6^ ^,^ ^11^ ^,^ ^17^ ^,^ ^22^ ^,^ ^23^ At the same time, inter-individual differences in microbial composition and gene content result in heterogeneous metabolic responses to similar dietary exposures, contributing to variability in circulating metabolite profiles and disease risk.^19^ ^,^ ^20^ ^,^ ^23^ ^,^ ^24^ This diet–microbe–host axis provides a unifying framework linking environmental exposures to microbial function and vascular pathology.

Importantly, these interconnected mechanisms are particularly evident in conditions characterized by chronic gut barrier disruption and immune activation. In this context, people with HIV represent a clinically relevant human model in which persistent microbial translocation, dysbiosis, and immune dysregulation converge to amplify microbiome-associated pathways of atherogenesis, providing translational insight into the gut–artery axis.^25-30^

In this Review, we synthesize current evidence linking the gut microbiome to atherosclerosis with a focus on microbial metabolites as central effectors of the gut–artery axis. We integrate multi-omic, mechanistic, and clinical data to (i) define the pathways connecting microbial metabolism to vascular disease, (ii) assess the strength of evidence supporting specific metabolites, and (iii) highlight translational opportunities and remaining challenges for advancing microbiome-based cardiovascular research.

## 2. Functional features of the gut microbiome associated with atherosclerosis

Although atherosclerosis-associated changes in gut microbial composition have been widely described, accumulating evidence indicates that alterations in microbial metabolic function—and the metabolites produced thereby—represent the most proximate link between the gut microbiome and vascular disease. Accordingly, this section emphasizes gut microbiome–associated functional features and microbial metabolites for which convergent epidemiological, experimental, and mechanistic evidence supports causality in atherogenesis.

The intestinal microbiota is a complex and metabolically active ecosystem dominated by the phyla *Firmicutes, Bacteroidetes, Actinobacteria, and Proteobacteria*, with substantial inter-individual variability driven by diet, host genetics, medications, and environment. A growing body of evidence from human cohorts, experimental models, and integrative multi-omic studies indicates that alterations in gut microbial composition and function are associated with atherosclerotic cardiovascular disease and contribute to its pathogenesis through effects on host metabolism, immune activation, and vascular function.

Rather than reflecting isolated taxonomic shifts, atherosclerosis-associated dysbiosis is increasingly understood as a functional reprogramming of the gut microbiome, characterized by loss of homeostatic, short-chain–fatty-acid (SCFA)–producing communities and enrichment of taxa and pathways that generate pro-inflammatory and pro-atherogenic metabolites.

### 2.1. Altered microbial diversity and composition

Early 16S rRNA gene sequencing and metagenomic studies demonstrated that individuals with coronary artery disease or carotid atherosclerosis exhibit reduced microbial *α*-diversity and distinct *β*-diversity compared with healthy controls^31^; however, these compositional changes are increasingly interpreted through their functional and metabolic consequences. Subsequent population-based and disease-specific cohorts have consistently reported compositional shifts marked by enrichment of facultative anaerobes and pathobionts—including *Enterobacteriaceae, Streptococcus, and Escherichia/Shigella*—and depletion of obligate anaerobic, butyrate-producing taxa such as *Faecalibacterium*, *Roseburia*, and *Agathobacter* ^10^ ^,^ ^11^ ^,^ ^32-34^ (Table 1).

**Table 1.** Microbial signatures associated with atherosclerosis.

Taxon or microbial function	Direction of association	Functional relevance	Atherogenic implication
*Streptococcus* spp.	↑	Histidine fermentation → ImP	Endothelial activation, mTORC1
*Ruminococcus gnavus*	↑	Mucin degradation, proteolytic metabolism	Systemic inflammation
*Enterobacteriaceae*	↑	LPS-rich outer membrane	TLR4 activation, IL-6/CRP elevation
*Faecalibacterium prausnitzii*	↓	Butyrate production	Anti-inflammatory, barrier integrity
cutC/D (TMA lyase genes)	↑	TMA generation	Platelet activation, foam cell formation
hutH/urdA (histidine-to-ImP pathway genes)	↑	Histidine → urocanate→ ImP	Accelerated plaque formation

Abbreviations: spp., Species; ImP, Imidazole propionate; mTORC1, Mechanistic target of rapamycin complex 1; LPS, Lipopolysaccharide; TLR4, Toll-like receptor 4; IL-6, Interleukin-6; CRP, C-reactive protein; TMA, Trimethylamine; cutC/D, Choline trimethylamine lyase genes; hutH, histidine ammonia-lyase gene; urdA, urocanate reductase gene.

Loss of butyrate-producing bacteria has important functional implications. Butyrate supports epithelial barrier integrity, suppresses pro-inflammatory signaling, and promotes regulatory immune responses via G-protein–coupled receptors (GPR41/43) and histone deacetylase inhibition.^35^ Depletion of these taxa is associated with increased intestinal permeability, microbial translocation, and systemic inflammation—processes central to atherogenesis.

Conversely, enrichment of *Streptococcus*, *Prevotella*, and *Ruminococcus gnavus* has been repeatedly observed in atherosclerosis and related cardiometabolic states.^10^ ^,^ ^34^ These taxa exhibit proteolytic and amino-acid–fermenting metabolic programs and have been implicated in the production of pro-atherogenic metabolites. Notably, several *Streptococcus* and *Prevotella* species harbor pathways for histidine catabolism leading to ImP production, a microbial metabolite now linked to vascular dysfunction and plaque development.^16^ ^,^ ^19^ ^,^ ^36^

Metagenomic analyses further support these observations. Enrichment of microbial genes involved in trimethylamine (TMA) synthesis (cutC/D, cntA/B) has been documented in individuals with atherosclerosis, indicating increased microbial capacity to generate atherogenic metabolites.^37^ While comprehensive enrichment of specific histidine/urocanate metabolism genes (e.g., hutH/urdA) has not been widely validated in humans, gut microbial metabolism of histidine-derived metabolites such as ImP has been linked to atherosclerosis progression and vascular dysfunction.^16^

Genetic epidemiology provides additional evidence consistent with causality. Mendelian randomization analyses using MiBioGen and FinnGen data suggest inverse associations between specific taxa (e.g., *Firmicutes* phylum, *Desulfovibrionaceae* family) and apolipoprotein B levels or coronary atherosclerosis risk, implying functional involvement of microbial communities in lipid metabolism and vascular disease rather than simple correlation.^38^ ^,^ ^39^

Interpretation of taxonomic associations in atherosclerosis is further complicated by substantial heterogeneity across studies. Reported differences in microbial composition often vary between cohorts, reflecting the influence of geography, habitual diet, age, sex, comorbidities, medication exposure, and technical factors including sequencing platforms, depth, and bioinformatic pipelines. These sources of variability can lead to inconsistent identification of specific taxa associated with atherosclerosis and limit reproducibility at the species level. Importantly, such heterogeneity does not necessarily imply lack of biological signal but rather highlights the context-dependent nature of host–microbiome interactions. In this setting, functionally convergent features—such as enrichment of pathways related to amino-acid fermentation, endotoxin production, and depletion of SCFA synthesis—appear more consistent across studies than taxonomic signatures alone. These observations support a shift toward pathway-level and metabolite-centric frameworks, which may provide more robust and generalizable insights into microbiome contributions to atherosclerosis, as developed in Section 2.2 and Section 3.

### 2.2. Functional signatures: metabolism, metabolites, and pathways

Beyond taxonomic composition, functional dysbiosis—defined by altered microbial metabolic output—appears central to atherosclerotic risk. Metagenomic, metatranscriptomic, and metabolomic studies consistently show upregulation of pathways related to lipopolysaccharide (LPS) biosynthesis, amino-acid fermentation, and bile-acid transformation in individuals with cardiovascular disease or subclinical atherosclerosis.^10^ ^,^ ^11^ ^,^ ^40^

Among these functional shifts, microbial pathways involved in dietary choline, carnitine, and amino-acid metabolism are repeatedly linked to circulating metabolites associated with atherosclerotic risk (Table 2). These include TMA–TMAO metabolism, phenylalanine-derived metabolites such as PAGln, and histidine-derived products including ImP, all of which show consistent associations with vascular dysfunction, thrombosis, or plaque burden across experimental and human studies.^6^ ^,^ ^14-18^ ^,^ ^41^

**Table 2.** Major studies linking gut microbiome–derived metabolites to cardiovascular disease.

Metabolite	Key study, reference	Study population/design	Main findings	Cardiovascular outcomes
Trimethylamine *N*-oxide (TMAO)	[14]	Human cohort and mechanistic mouse studies	Gut microbiota metabolize dietary choline to TMAO; higher circulating levels associated with atherosclerosis	Angiographic measures of coronary artery atherosclerotic burden and cardiac risks; experimental atherosclerosis
	[42]	Prospective cohort study	Plasma TMAO levels predicted major adverse cardiovascular events	Higher risk of myocardial infarction, stroke, and death
	[17]	Human and animal study	Microbial metabolism of L-carnitine generates TMAO	Increased risks for prevalent CVD and incident MACEs with high TMAO; promotion of atherosclerosis
	[43–45]	Human cohorts with vascular imaging	Increased cIMT, higher CAC scores, and greater coronary plaque burden in asymptomatic and at-risk population	Plasma TMAO concentrations associate with subclinical atherosclerosis and atherosclerotic burden
Phenylacetylglutamine (PAGln)	[15]	Human cohort and mechanistic experiments	PAGln derived from microbial phenylalanine metabolism activates adrenergic receptors on platelets	Enhanced platelet activation and thrombosis risk
	[46,47]	Human cohorts with vascular imaging	Higher arterial stiffness and more severe coronary atherosclerosis by CCTA	Higher circulating PAGln correlates with subclinical atherosclerosis
Imidazole propionate (ImP)	[16,19]	Human and mechanistic experimental study	Histidine-derived microbial metabolite activates mTORC1 signaling	Metabolic and endothelial dysfunction, vascular inflammation, and accelerated plaque formation in experimental models
	[16]	Human cohorts with vascular imaging	Associations with total plaque burden, CAC and metabolically active plaque with FDG-PET	Circulating ImP levels associated with subclinical atherosclerosis
Short-chain fatty acids (SCFAs) and bile acids	[48–52]	Human cohort and animal studies	SCFAs: improved barrier function, anti-inflammatory, immune modulation. Secondary bile acids: activate TGR5/FXR → inhibit NF-κB and NLRP3, modulate mitochondrial ROS, activate cAMP–PKA signaling	Potential protective effects against cardiometabolic disease

Abbreviations: TMAO, trimethylamine *N*-oxide; TMA, trimethylamine; CVD, cardiovascular disease; MACE(s), major adverse cardiovascular event(s); cIMT, carotid intima–media thickness; CAC, coronary artery calcium; CCTA, coronary computed tomography angiography; FDG-PET, fluorodeoxyglucose positron emission tomography; PAGln, phenylacetylglutamine; ImP, imidazole propionate; SCFAs, short-chain fatty acids; mTORC1, mechanistic target of rapamycin complex 1; TGR5, Takeda G protein–coupled receptor 5; FXR, farnesoid X receptor; NLRP3, NOD-like receptor family pyrin domain containing 3 inflammasome; cAMP–PKA, cyclic AMP–protein kinase A; ROS, reactive oxygen species; NF-κB, nuclear factor kappa B.

In parallel, atherosclerosis-associated microbiomes are characterized by depletion of pathways involved in SCFA production, particularly butyrate and propionate.^9^ Given the established roles of SCFAs in maintaining epithelial barrier integrity, immune homeostasis, and vascular protection, loss of these metabolic functions may further predispose to systemic inflammation and endothelial dysfunction.^13^ ^,^ ^48^

Importantly, microbial metabolic capacity reflects not only taxonomic composition but also functional redundancy across the ecosystem. Distinct microbial taxa can encode overlapping enzymatic pathways and thereby generate similar metabolic outputs, such that comparable profiles of bioactive metabolites may arise from divergent community structures. This functional redundancy helps explain the limited reproducibility of taxonomic associations across cohorts and underscores the importance of pathway-level and metabolite-centric analyses. At the same time, ecological interactions—including cross-feeding, substrate competition, and niche specialization—further shape metabolic flux, such that metabolite production represents an emergent property of the microbial community rather than the activity of individual taxa alone. Together, these features support a shift from taxonomic to functional interpretation of the microbiome and reinforce the rationale for focusing on microbial metabolites as integrative readouts of host–microbiome interactions, as developed in Section 3.

### 2.3. Determinants of microbial metabolic capacity

Diet represents the principal upstream determinant of microbial metabolic capacity, governing the availability of substrates that fuel microbial biochemical pathways and, ultimately, the generation of bioactive metabolites. Nutrients such as choline, L-carnitine, aromatic amino acids, and complex carbohydrates are differentially metabolized by the gut microbiota into compounds with distinct vascular and immunometabolic effects, including TMA, phenylacetic acid derivatives, indole metabolites, and SCFAs.^14-19^ Both long-term dietary patterns and short-term dietary exposures are known to influence microbial metabolic output, although sustained dietary habits appear to exert the dominant effect on microbial functional configuration.^6^ ^,^ ^11^ Importantly, substantial inter-individual variability exists in metabolite production in response to similar dietary inputs, reflecting differences in microbial composition, gene content, and enzymatic capacity.^19^ ^,^ ^21^ These observations support a diet–microbe–host axis in which dietary exposures shape microbial metabolism and thereby modulate downstream pathways relevant to atherosclerosis, including barrier integrity, immune activation, lipid handling, and thrombosis. As discussed in Section 3, these diet-dependent metabolic pathways give rise to key metabolite classes—such as TMAO, PAGln, and ImP—that link the gut microbiome to vascular disease.

Host genetics also shape microbial composition. Genome-wide association studies have identified variants at loci such as *LCT* that influence the abundance of taxa including *Bifidobacterium*, highlighting gene–diet–microbiome interactions.^23^ Medications—including antibiotics, proton pump inhibitors, statins, and metformin—further modulate microbial structure and metabolic output and represent important confounders in observational studies.^53^ ^,^ ^54^

Additional factors such as age, sex, comorbidities, and lifestyle contribute to inter-individual variability. Emerging evidence also supports crosstalk between the oral and gut microbiomes in atherosclerosis, suggesting that microbial communities beyond the intestine may influence vascular disease through translocation or shared inflammatory pathways.^55^

Collectively, these determinants converge on microbial function rather than taxonomy alone. Atherosclerosis is consistently associated with reduced microbial diversity, depletion of SCFA producing taxa, and enrichment of proteolytic and amino acid–fermenting bacteria, a configuration that favors production of metabolites such as TMAO, PAGln and ImP. This functional framework provides a mechanistic bridge between microbial ecology and vascular disease and sets the stage for the metabolite-centered discussion in the following section.

## 3. Mechanistic conduits from gut to plaque

**Figure 1.**
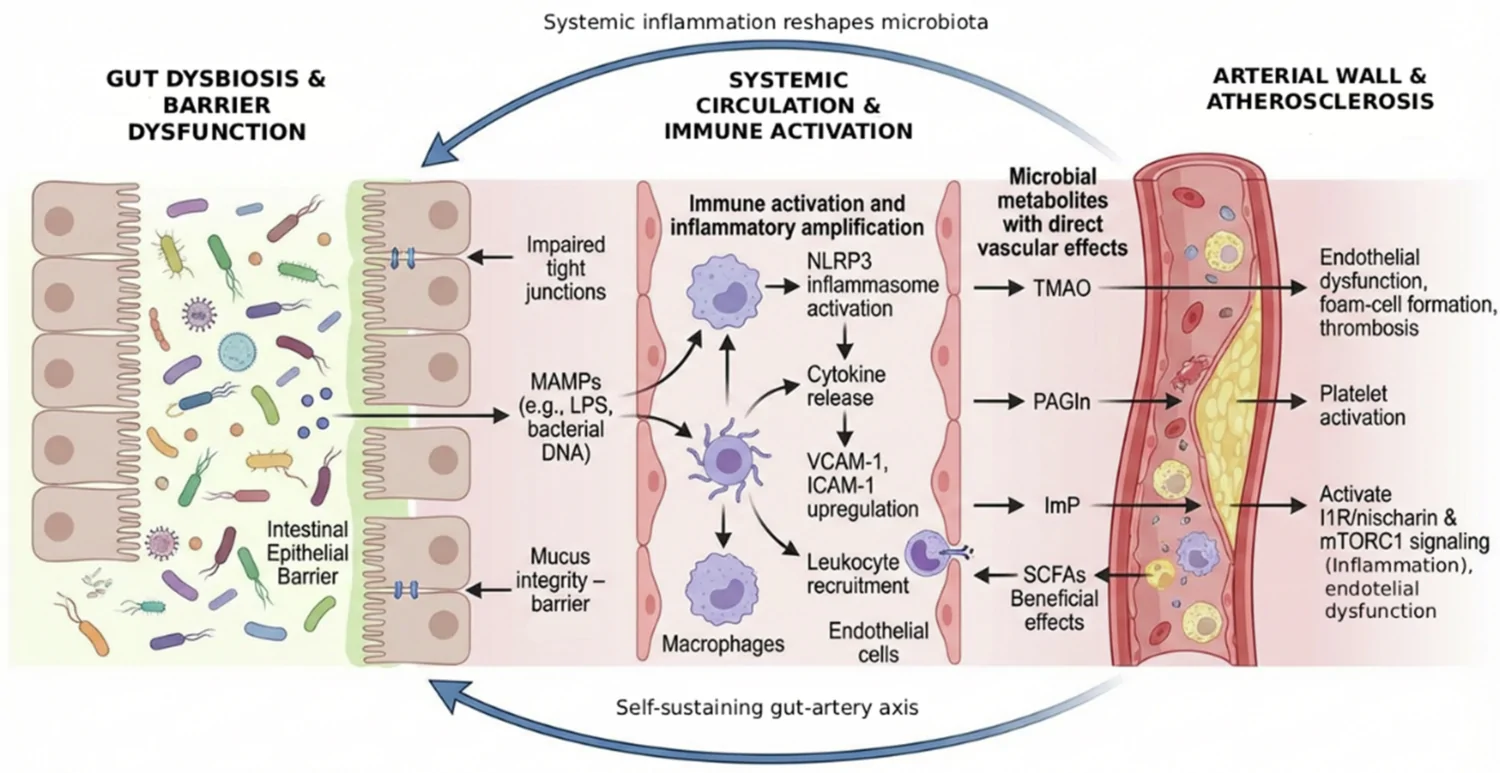
Mechanistic conduits linking the gut microbiome to atherosclerosis. The schematic illustrates three interrelated mechanisms through which gut microbial dysbiosis promotes atherogenesis. (1) Intestinal barrier dysfunction and microbial translocation: Dysbiosis, diet, aging, and medications impair epithelial tight junctions and mucus integrity, increasing gut permeability and permitting translocation of microbial-associated molecular patterns (MAMPs), including lipopolysaccharide (LPS), peptidoglycan, and bacterial DNA. These signals activate pattern-recognition receptors (e.g., TLR4, NOD1/2) on endothelial and innate immune cells, inducing NF-κB signaling, cytokine release, and upregulation of adhesion molecules (VCAM-1, ICAM-1), thereby promoting leukocyte recruitment to the arterial wall. (2) Immune activation and inflammatory amplification: Chronic exposure to microbial products reprograms macrophages toward a pro-inflammatory phenotype, activates the NOD-, LRR- and pyrin domain-containing protein 3 (NLRP3) inflammasome, and disrupts endothelial nitric oxide signaling. Systemic inflammation feeds back to reshape gut microbial ecology, enriching facultative anaerobes and sustaining dysbiosis. (3) Microbial metabolites with direct vascular effects: Gut-derived metabolites exert direct effects on vascular and immune cells. Trimethylamine *N*-oxide (TMAO) promotes endothelial dysfunction, foam-cell formation, and thrombosis; phenylacetylglutamine (PAGln) enhances platelet activation via adrenergic receptors; imidazole propionate (ImP) activates the imidazoline-1 receptor (I1R/nischarin) and mTORC1 signaling, driving oxidative stress and inflammatory responses. Beneficial metabolites such as short-chain fatty acids (SCFAs) strengthen barrier integrity and exert anti-inflammatory effects. Together, these pathways establish a self-sustaining gut–artery axis promoting atherosclerosis. This signaling is bidirectional: systemic inflammation reshapes gut ecology, reinforcing dysbiosis and sustaining microbial production of atherogenic metabolites.

Microbiome-mediated effects on atherogenesis converge on three interconnected mechanistic axes (Figure 1): (i) disruption of intestinal barrier integrity with microbial translocation; (ii) immune activation and inflammatory amplification; and (iii) production of bioactive microbial metabolites that directly modulate vascular, immune, and platelet function. These pathways operate in parallel and reinforce one another, establishing a self-sustaining gut–artery axis that promotes endothelial dysfunction, plaque formation, and atherothrombosis (Table 3).

**Table 3.** Mechanistic conduits linking the gut microbiome to endothelial dysfunction and atherosclerotic plaque development.

Mechanistic axis	Gut-derived trigger(s)	Key molecular/cellular pathways	Vascular and plaque-level consequences
Intestinal barrier dysfunction and microbial translocation	Dysbiosis; dietary excess; aging; inflammatory stress → ↑ intestinal permeability; systemic dissemination of MAMPs (e.g., LPS, peptidoglycan, bacterial DNA)	Activation of endothelial and myeloid pattern-recognition receptors (TLR4, NOD-like receptors); NF-κB signaling; cytokine (IL-6, TNF-*α*) and adhesion molecule (VCAM-1, ICAM-1) induction; eNOS inhibition; mitochondrial ROS generation	Endothelial dysfunction; impaired nitric oxide bioavailability; leukocyte adhesion and transmigration; initiation and acceleration of plaque formation; vascular aging
Immune activation and inflammatory amplification	Persistent exposure to translocated microbial products and pro-inflammatory microbial cues	Monocyte/macrophage reprogramming; NLRP3 inflammasome activation; IL-1β and IL-18 secretion; impaired cholesterol handling; inflammation-driven reshaping of gut ecology (oxygenation, nitrate availability)	Sustained arterial inflammation; enhanced foam-cell formation; plaque growth and instability; feed-forward gut–vascular inflammatory loop
Microbial metabolic signaling (integrated, axis-level effects)	Chronic exposure to bioactive microbial metabolites (summarized in Table 2)	Direct modulation of endothelial, immune, and platelet signaling; suppression of eNOS activity; oxidative stress; inflammasome engagement; platelet hyperreactivity; interaction with host metabolic and stress-response pathways	Endothelial activation; impaired vasodilation; increased thrombogenicity; accelerated plaque progression and atherothrombotic risk
Host–microbiome feedback reinforcement	Systemic inflammation altering epithelial and microbial ecology	NF-κB–dependent transcriptional and proteomic reprogramming; reciprocal shaping of gut microbiota composition and function	Self-sustaining gut–artery axis; persistence of vascular inflammation; promotion of plaque vulnerability and adverse cardiovascular outcomes

Abbreviations: BA, Bile acids; eNOS, Endothelial nitric oxide synthase; HDAC, Histone deacetylase; ICAM-1, Intercellular adhesion molecule-1; IL-1β, Interleukin-1 beta; IL-6, Interleukin-6; LPS, Lipopolysaccharide; MAMPs, Microbial-associated molecular patterns; mTORC1, Mechanistic target of rapamycin complex 1; NF-κB, Nuclear factor kappa-light-chain-enhancer of activated B cells; NLRP3, NOD-, LRR-, and pyrin domain-containing protein 3; PRRs, Pattern-recognition receptors; ROS, Reactive oxygen species; TLR4, Toll-like receptor 4; TNF-*α*, Tumor necrosis factor alpha; VCAM-1, Vascular cell adhesion molecule-1.

Among these, microbial metabolites represent the most direct and experimentally tractable mediators, providing a mechanistic bridge between microbial ecology and vascular pathology. Importantly, as outlined in Section 2.3, their generation is fundamentally constrained by dietary substrate availability and microbial functional capacity, such that inter-individual variation in diet–microbiome interactions directly shapes circulating metabolite profiles and downstream vascular effects.^11^ ^,^ ^13-19^ ^,^ ^21^ The following sections delineate the complementary roles of barrier dysfunction and immune activation, which together establish the biological context in which metabolite-driven mechanisms operate.

### 3.1. Intestinal barrier dysfunction and microbial translocation

The intestinal epithelium constitutes a critical interface between the luminal microbiota and the systemic circulation. Under homeostatic conditions, tight junctions, mucus layers, antimicrobial peptides, and immune surveillance mechanisms collectively limit the translocation of microbial products. Disruption of this barrier—driven by dysbiosis, dietary patterns, aging, and inflammatory stress—increases intestinal permeability and permits systemic dissemination of microbial-associated molecular patterns (MAMPs), including LPS, peptidoglycan, and bacterial DNA.^56^ ^,^ ^57^

Circulating microbial products activate pattern-recognition receptors on endothelial cells and innate immune cells, inducing inflammatory signaling pathways that promote leukocyte recruitment and early atherogenic responses.^58^ ^,^ ^59^ Chronic low-grade endotoxaemia, reflected by elevated plasma LPS or LPS-binding protein, has been associated with endothelial dysfunction, insulin resistance, carotid plaque burden, and cardiovascular mortality in human studies.^60^ ^,^ ^61^

At the vascular level, experimental studies demonstrate that physiologically relevant LPS concentrations impair endothelial nitric oxide synthase (eNOS) activity, increase mitochondrial reactive oxygen species, and disrupt endothelial bioenergetics, thereby directly promoting endothelial activation and dysfunction.^62^ ^,^ ^63^ Age-related reductions in epithelial tight-junction proteins further exacerbate gut permeability, linking barrier decline to systemic inflammation and vascular aging.^64^ ^,^ ^65^

Beyond monocytes and macrophages, neutrophils, T cells, and B cells also contribute to microbiome-associated vascular inflammation. These immune populations influence plaque development through effects on innate and adaptive immune responses, thereby modulating cardiovascular risk.^66-71^

This mechanism is particularly evident in people with HIV, in whom early depletion of gut-associated CD4⁺ and Th17 cells leads to persistent mucosal injury and microbial translocation despite effective antiretroviral therapy.^25^ ^,^ ^26^ Elevated levels of LPS and soluble CD14 predict cardiovascular events in this population, underscoring the clinical relevance of sustained barrier dysfunction as a driver of vascular risk.^27^

### 3.2. Immune activation and inflammatory amplification

Microbial translocation and metabolite exposure converge to reprogram innate and adaptive immune responses in ways that promote atherogenesis. Chronic stimulation of monocytes and macrophages by microbial products induces a pro-inflammatory phenotype characterized by increased cytokine production, inflammasome activation, and impaired cholesterol handling, thereby facilitating foam cell formation and plaque progression.

The NLRP3 (NOD-, LRR- and pyrin domain-containing protein 3) inflammasome occupies a central position in this process. Both microbial components and selected microbial metabolites activate NLRP3 signaling in vascular and immune cells, leading to secretion of IL-1β and IL-18 and amplification of local and systemic inflammation.^18^ Endothelial cells exposed to these stimuli upregulate adhesion molecules and chemokines, further promoting monocyte recruitment and vascular inflammation.

Adaptive immune responses further amplify inflammatory signaling initiated by microbial translocation and metabolite exposure, contributing to a sustained pro-atherogenic immune environment.

Importantly, immune activation feeds back to reshape the gut ecosystem itself. Inflammatory signaling alters epithelial metabolism, increases luminal oxygenation and nitrate availability, and creates ecological niches favoring facultative anaerobes such as *Enterobacteriaceae* at the expense of obligate anaerobes.^72^ ^,^ ^73^ This inflammation-driven ecological shift reinforces dysbiosis and sustains microbial production of pro-inflammatory molecules, establishing a feed-forward loop linking gut inflammation to vascular injury.

### 3.3. Microbial metabolites as central effectors of the gut–artery axis

Microbial metabolites represent the most direct functional interface between the gut microbiome and host vascular biology. By integrating dietary substrates, microbial enzymatic activity, and host signaling pathways, these small molecules translate microbial metabolic capacity into systemic effects on endothelial function, immune activation, lipid metabolism, and thrombosis. This metabolite-centered perspective provides a mechanistic framework that extends beyond taxonomic associations and enables more precise interrogation of microbiome–host interactions in atherosclerosis.

Major classes of microbiota-derived metabolites with potential relevance to atherosclerosis include SCFA produced through fermentation of dietary fiber; secondary bile acids generated through microbial transformation of primary bile acids; tryptophan-derived metabolites such as indole derivatives including indole propionate (IPA); and amino acid– and nutrient-derived metabolites such as TMAO, PAGln, and ImP. However, while many microbial metabolites have been associated with cardiometabolic disease, only a subset currently meets criteria consistent with a causal role in atherosclerosis. To distinguish association from causality and to prioritize metabolites with potential mechanistic and translational relevance, we applied the set of criteria summarized in Box 1.

Box 1. From association to causality: criteria for prioritizing microbial metabolites in atherosclerosis.

- **Reproducible association across cohorts**
  Consistent links between circulating metabolite levels and subclinical or clinical atherosclerosis in independent populations.
- **Defined microbial and dietary origins**
  Identification of microbial taxa, genes, or pathways responsible for metabolite production, and their dietary precursors.
- **Mechanistic activity in vascular or immune cells**
  Demonstrated effects on endothelial function, macrophage lipid handling, platelet activation, or inflammatory signaling.
- **Engagement of host receptors or signaling pathways**
  Evidence for specific molecular targets (e.g., GPCRs, nuclear receptors, mTORC1, adrenergic signaling).
- **Causal evidence from experimental models**
  Gain- or loss-of-function studies demonstrating direct effects on atherosclerotic plaque development, progression, or vascular inflammation.
- **Modifiability and translational potential**
  Sensitivity to dietary intervention, microbial modulation, or pharmacologic targeting.

Guided by these criteria, this section focuses on metabolites for which convergent epidemiological, mechanistic, and experimental evidence supports a role in atherogenesis. Rather than providing an exhaustive catalog, we prioritize representative pathways that illustrate how microbial metabolism interfaces with vascular and immunometabolic processes, while acknowledging that many metabolites act within interconnected and context-dependent networks. While SCFAs, bile acids, and tryptophan-derived metabolites appear to play modulatory or protective roles in cardiometabolic health, three metabolites, namely TMAO, PAGln, and ImP, currently have the most consistent evidence supporting a direct mechanistic contribution to atherogenesis. These metabolites are therefore discussed in detail below.

Importantly, several of the pathways discussed below are also implicated in broader cardiometabolic disorders, including obesity, insulin resistance, and metabolicdysfunction-associated steatotic liver disease. In this context, microbial metabolites may function both as systemic mediators of metabolic inflammation and as more specific modulators of vascular pathology, depending on their downstream targets and tissue-specific effects. Distinguishing between these roles is essential for interpreting their relevance as biomarkers or therapeutic targets in atherosclerosis.

#### 3.3.1. Trimethylamine N-oxide (TMAO)

Trimethylamine N-oxide is generated from dietary choline, carnitine, and betaine via microbial formation of TMA and hepatic oxidation by flavin monooxygenase 3 (FMO3). TMAO is one of the most extensively characterized microbial metabolites in atherosclerosis. Extensive experimental evidence demonstrates that TMAO promotes atherosclerosis by enhancing macrophage foam-cell formation, impairing reverse cholesterol transport, activating the NLRP3 inflammasome, and increasing platelet hyperreactivity.^14^ ^,^ ^17^ ^,^ ^18^ ^,^ ^74^

TMAO also reduces endothelial nitric oxide bioavailability by suppressing eNOS activity and increasing oxidative stress, thereby contributing to endothelial dysfunction.^75^ Inhibition of microbial TMA formation or liver-specific knockdown of FMO3 markedly attenuates atherosclerotic lesion development in murine models, supporting a causal role.^41^ ^,^ ^76^

Clinically, elevated circulating TMAO predicts major adverse cardiovascular events,^42^. However, inter-individual variability related to renal function, diet, and host genetics modulates circulating levels and effect sizes, emphasizing the importance of contextual interpretation.

According to the criteria outlined in Box 1, TMAO represents one of the most extensively supported microbiome-derived metabolites, with consistent epidemiological associations, well-characterized mechanistic pathways, and robust experimental validation across multiple model systems. While residual uncertainties remain, the overall evidence supports a contributory role in atherothrombotic disease.

#### 3.3.2. Phenylacetylglutamine (PAGln)

Phenylacetylglutamine is produced through microbial conversion of phenylalanine to phenylacetic acid, followed by hepatic conjugation with glutamine. PAGln enhances platelet activation and thrombosis by signaling through α₂A-, α₂B-, and β₂-adrenergic receptors.^15^ Genetic variation in adrenergic receptor pathways modifies PAGln-associated risk, strengthening causal inference.

Elevated PAGln levels associate with platelet hyperreactivity, arterial stiffness, and increased incidence of cardiovascular events independently of traditional risk factors.^6^ ^,^ ^15^ ^,^ ^46^ These findings identify PAGln as a gut-derived, catecholamine-mimicking metabolite linking microbial amino-acid metabolism to thrombosis and vascular risk.

Within the Box 1 framework, PAGln is supported by strong epidemiological and mechanistic evidence, particularly linking microbial metabolism to platelet hyperreactivity and thrombotic risk, although experimental validation remains more limited compared with TMAO. These features position PAGln as a biologically plausible mediator of cardiovascular risk, with growing but still evolving causal support.

#### 3.3.3. Imidazole propionate (ImP)

Imidazole propionate (ImP) is a histidine-derived microbial metabolite that has recently emerged as a potential link between gut microbiome function and cardiometabolic disease.^16^ ^,^ ^19^ ^,^ ^36^ Elevated circulating levels of ImP have been reported in individuals with metabolic disorders^19^ and, more recently, in association with subclinical atherosclerosis and vascular inflammation.^21^ ^,^ ^77^ Moreover, elevated circulating ImP levels have been associated with an increased risk of adverse cardiovascular events in patients with coronary artery disease, independent of traditional risk factors.^78^

Mechanistically, ImP has provided important insights into receptor-mediated microbiome–host signaling. Experimental studies demonstrate that ImP activates the imidazoline-1 receptor, leading to downstream activation of mTORC1 signaling pathways implicated in endothelial dysfunction, inflammation, and metabolic dysregulation.^36^ ^,^ ^77^ In preclinical models, ImP impairs endothelial function and promotes several processes relevant to atherogenesis, supporting biological plausibility for a role in vascular pathology.

However, current evidence remains primarily derived from in vitro systems, animal models, and cross-sectional or associative human studies. Longitudinal data, interventional studies, and direct modulation of ImP pathways in humans are still lacking. In addition, Mendelian randomization analyses and other approaches capable of strengthening causal inference have not yet been established for this pathway. Accordingly, careful interpretation is required to avoid overextension of causal claims.

Within the Box 1 framework, ImP fulfills several important mechanistic and experimental criteria, including a defined receptor-mediated signaling pathway. However, evidence in humans remains largely observational. Accordingly, ImP should currently be regarded as a promising candidate mediator of atherosclerosis rather than a definitively established causal factor.

#### 3.3.4. Other microbial metabolites

In contrast to TMAO, PAGln, and ImP, which are generally associated with pro-atherogenic effects, other microbiota-derived metabolites appear to exert protective or regulatory effects on host metabolism and vascular biology. These include SCFAs and tryptophan-derived metabolites such as IPA, as well as secondary bile acids that interact with host metabolic and inflammatory pathways. Although these metabolites may not fulfill all criteria for direct causality in atherosclerosis, they contribute to intestinal and metabolic homeostasis, and reduced production may favor a pro-atherogenic environment.

Tryptophan-derived microbial metabolites, particularly IPA, have also emerged as potential modulators of cardiometabolic health. Higher circulating IPA levels have been associated with reduced risk of type 2 diabetes, improved intestinal barrier function, and lower systemic inflammation, and recent observational and experimental data suggest links with subclinical atherosclerosis and vascular dysfunction.^79-82^ Experimental studies further indicate that IPA may influence macrophage cholesterol handling and reverse cholesterol transport, supporting biological plausibility for a role in atherosclerosis.^80^ Although current evidence supports a protective cardiometabolic profile, further mechanistic and longitudinal studies are needed to determine whether IPA plays a direct causal role in atherogenesis.

Short-chain fatty acids, including acetate, propionate, and butyrate, exert vasculoprotective effects by strengthening epithelial barrier integrity, suppressing inflammation, and modulating vascular tone through GPR41/43 signaling and histone deacetylase inhibition.^48-50^ Propionate supplementation reduces atherosclerotic lesion formation and systemic inflammation in experimental models, partly via regulatory T-cell expansion.^13^

Secondary bile acids generated by microbial transformation of primary bile acids interact with host receptors such as FXR and TGR5, influencing lipid metabolism, inflammation, and endothelial activation.^51^ ^,^ ^83^ Activation of TGR5 suppresses NF-κB signaling and endothelial adhesion molecule expression, whereas FXR deficiency exacerbates atherosclerosis in experimental models.^52^ ^,^ ^84^

Within the Box 1 framework, SCFAs, IPA, and bile acids fulfill several mechanistic and experimental criteria but display complex, context-dependent effects that limit straightforward causal classification in atherosclerosis. These features underscore the importance of considering metabolic networks and host context when interpreting microbiome-derived metabolites.

#### 3.3.5. Integrative perspective: metabolite networks and host response

Although individual metabolites provide valuable mechanistic insights, it is increasingly clear that microbiome–host interactions operate through integrated metabolic networks rather than isolated pathways. Multi-omic studies combining metagenomics, metabolomics, and proteomics have identified coordinated gene–metabolite–host protein signatures associated with subclinical and clinical atherosclerosis.^20^ ^,^ ^85^ ^,^ ^86^ These analyses reveal that microbial metabolites interact with host pathways in a highly interconnected and context-dependent manner.

Moreover, functional redundancy within the microbiome means that similar metabolic outputs can be generated by different microbial communities, further emphasizing the importance of focusing on function rather than taxonomy alone. Inter-individual variability in diet, microbial composition, and host response adds an additional layer of complexity, shaping both metabolite production and downstream biological effects.

Taken together, these observations support a model in which microbial metabolites act as central but context-dependent effectors within a broader gut–artery axis. Integrating metabolite-specific insights with systems-level approaches will be essential to refine causal inference, identify clinically relevant biomarkers, and develop targeted therapeutic strategies.

### 3.4. Integrated host–microbiome crosstalk

Microbial metabolites and translocated products rewire host proteomic and transcriptomic networks that propagate vascular inflammation. Multi-omic studies demonstrate that exposure to TMAO or ImP upregulates NF-κB–dependent cytokines, adhesion molecules, and acute-phase proteins—molecular signatures mirrored in plasma proteomic profiles associated with plaque burden and instability.^20^ ^,^ ^21^

This signaling is bidirectional: systemic inflammation reshapes gut ecology, reinforcing dysbiosis and sustaining microbial production of atherogenic metabolites. Together, these processes establish a self-reinforcing inflammatory circuit linking gut barrier dysfunction, immune activation, microbial metabolism, and arterial disease.

Importantly, many of the pathways described—microbial metabolite production, immune activation, and metabolic inflammation—are shared across cardiometabolic diseases, including obesity and metabolic dysfunction–associated steatotic liver disease. While these mechanisms are not specific to atherosclerosis, their downstream consequences manifest in a vascular-specific manner, including endothelial dysfunction, plaque formation, and thrombosis. In this context, microbial metabolites may act both as systemic mediators of cardiometabolic risk and as context-dependent modulators of vascular pathology, rather than exclusively as disease-specific drivers.

**Figure 2.**
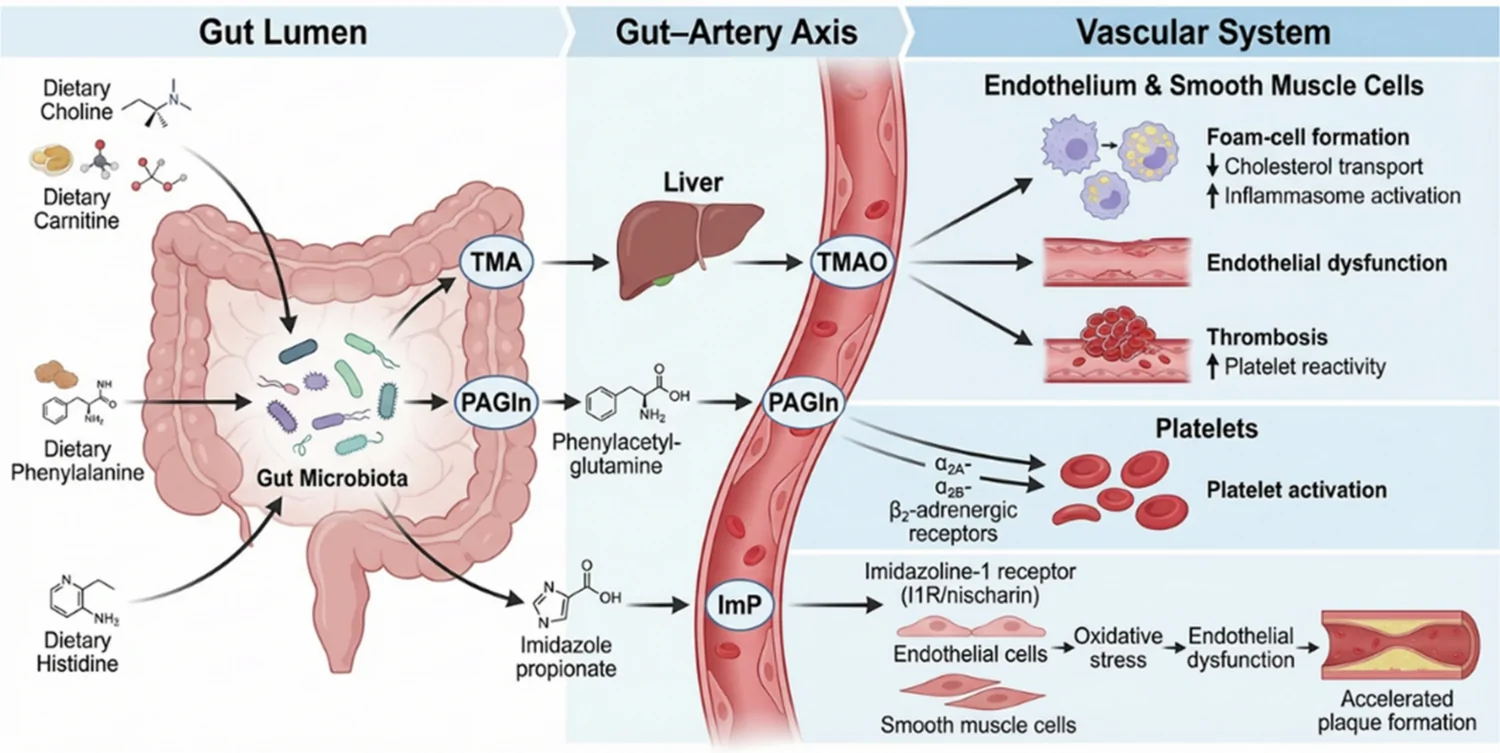
Microbial metabolites and host receptors with vascular effects. Major gut microbiota–derived metabolites influencing vascular biology. Schematic overview of key microbial metabolites implicated in atherosclerosis and their principal host targets. TMAO, generated from dietary choline and carnitine, promotes foam-cell formation, endothelial dysfunction, and thrombosis through effects on reverse cholesterol transport, inflammasome activation, and platelet reactivity. PAGln, derived from microbial phenylalanine metabolism, enhances platelet activation via α₂A-, α₂B-, and β₂-adrenergic receptors. Imidazole propionate (ImP), produced through microbial histidine fermentation, activates the imidazoline-1 receptor (I1R/nischarin), leading to mTORC1 signaling, oxidative stress, endothelial dysfunction, and accelerated plaque formation. Abbreviations: TMAO, Trimethylamine *N*-oxide; PAGln, Phenylacetylglutamine; ImP, Imidazole propionate; I1R, Imidazoline-1 receptor.

In summary, gut microbiome-atherosclerosis interactions emerge from coordinated effects on barrier function, immune regulation, and microbial metabolism. Among microbial metabolites, TMAO, PAGln, and ImP currently have the strongest mechanistic support for involvement in atherosclerosis, although the strength of causal evidence differs across metabolites as discussed in Section 3.3. These convergent pathways form a self-sustaining gut–artery axis that integrates microbial ecology with vascular pathology and provides a conceptual framework for biomarker discovery and therapeutic intervention. Figure 2 summarizes the direct effects of gut microbial metabolites on atherosclerosis.

## 4. Evidence map: subclinical and clinical atherosclerosis

A growing body of evidence from population cohorts, advanced vascular imaging, and prospective outcome studies supports a substantive link between gut microbial composition, circulating microbial metabolites, and atherosclerotic cardiovascular disease. Although many associations remain observational, convergence across vascular imaging, metabolomics, and mechanistic experimentation strengthens biological plausibility and supports a causal gut–vascular axis.

### 4.1. Subclinical atherosclerosis: imaging-based evidence

Carotid intima–media thickness (cIMT), coronary artery calcium (CAC), and multimodal plaque imaging are established surrogate markers of atherosclerotic burden and predictors of future cardiovascular events.^87^ ^,^ ^88^ Several cohort studies have linked gut-derived metabolites and microbial features to these subclinical phenotypes.

Plasma TMAO concentrations associate with increased cIMT, higher CAC scores, and greater coronary plaque burden in asymptomatic and at-risk populations^43-45^ (Table 2). These associations persist after adjustment for traditional cardiovascular risk factors and renal function in some cohorts, although effect sizes vary across populations.

Phenylacetylglutamine has been linked to subclinical vascular phenotypes through its pro-thrombotic properties. Higher circulating PAGln correlates with arterial stiffness and more severe coronary atherosclerosis assessed by coronary computed tomography angiography (CCTA).^46^ ^,^ ^47^

Imidazole propionate has emerged as a robust biomarker of early, metabolically active atherosclerosis. In the PESA cohort, a large population-based imaging study combining carotid ultrasound, coronary computed tomography, and multi-territorial plaque assessment, Mastrangelo et al.^16^ demonstrated that circulating ImP levels were significantly higher in asymptomatic individuals with subclinical atherosclerosis. ImP showed both linear and non-linear associations with total plaque burden and CAC and improved discrimination of early disease beyond LDL-cholesterol and high-sensitivity C-reactive protein (hs-CRP). These findings were independently replicated in a second asymptomatic cohort (IGT cohort, n = 1,844), confirming the reproducibility of the association.^16^

Importantly, ImP was also linked to metabolically active plaque. Using fluorine‑18 fluorodeoxyglucose positron emission tomography (18F-FDG PET) imaging, individuals with FDG-positive arterial lesions exhibited higher circulating ImP concentrations, even after adjustment for traditional risk factors. Associations were strongest in participants with concomitant bone marrow activation and systemic inflammatory signatures, suggesting that ImP identifies inflammatory plaque phenotypes rather than calcified disease alone.^16^ Collectively, multimodal imaging studies place ImP at the intersection of microbial metabolism and inflammatory plaque activity.

Beyond metabolites, gut microbial composition itself associates with vascular structure. Enrichment of *Streptococcus* spp. and *Ruminococcus gnavus*—taxa implicated in histidine fermentation and ImP production— correlated with > 50% stenosis in single or multiple vessels on coronary angiography, whereas butyrate-producing taxa such as *Faecalibacterium prausnitzii* and *Roseburia intestinalis* were depleted.^10^ In longitudinal studies of people with HIV, distinct gut microbial signatures predicted cIMT progression and incident carotid plaque^30^ (Table 4).

**Table 4.** Studies linking microbial profile/metabolites to cardiovascular disease in people with HIV.

First author (Ref.)	Population/sample size	Study design	Microbial feature/metabolite	Cardiovascular outcome	Main findings
Masiá M et al. [30]	Virally suppressed PWH on ART/*n* = 167	Prospective longitudinal cohort study	Enrichment of *Bilophila, Ruminococcus 2*	cIMT progression	Pro-inflammatory genera independently predicted cIMT progression
Srinivasa S et al. [89]	ART-treated PWH undergoing coronary CTA/*n* = 155	Cross-sectional imaging study	Trimethylamine (TMA), TMAO	CAC score, calcified plaque segments, total plaque volume	TMA (not TMAO) independently associated with CAC and plaque burden
Shan Z et al. [90]	Two longitudinal HIV cohorts/*n* = 520	Prospective	TMAO	Carotid plaque (cIMT)	Baseline TMAO predicted incident carotid plaque progression
Missailidis C et al. [91]	HIV cohort on chronic ART/*n* = 101	Cross-sectional, retrospective	TMAO	Cardiovascular events, inflammatory markers	No association with inflammatory markers or cardiovascular events
García-Abellán J et al. [92]	ART-treated PWH/*n* = 169	Observational cohort	*Tyzzerella_4, Dialister, Lactobacillus*	Metabolic phenotype linked to vascular risk	ART-associated microbial shifts paralleled adverse cardiometabolic phenotypes
Trøseid M et al. [93]	Copenhagen HIV cohort/*n* = 254	Cross-sectional	Imidazole propionate (ImP)	Obstructive CAD	Dysbiosis associated with CAD; ImP not independently associated

Abbreviations: PWH, people with HIV; ART, antiretroviral therapy; CTA, computed tomography angiography; TMA, trimethylamine; TMAO, trimethylamine *N*-oxide; CAC, coronary artery calcium; cIMT, carotid intima–media thickness; ImP, imidazole propionate; CAD, coronary artery disease; hsCRP, high-sensitivity C-reactive protein; sCD14, soluble CD14; LPS, lipopolysaccharide.

### 4.2. Clinical cardiovascular outcomes

Large prospective cohorts have consistently linked microbial metabolites to incident cardiovascular events. Elevated circulating TMAO predicts myocardial infarction, stroke, heart failure, and cardiovascular mortality independently of traditional risk factors.^17^ ^,^ ^42^ Similar associations have been observed in chronic kidney disease cohorts, highlighting the interaction between renal clearance, microbial metabolism, and cardiovascular risk.^94^ ^,^ ^95^

Phenylacetylglutamine has also demonstrated prognostic value. In prospective analyses, higher PAGln concentrations predicted major adverse cardiovascular events and thrombosis-related outcomes, consistent with its mechanistic effects on platelet activation.^15^

Imidazole propionate has been validated as a clinically relevant metabolite associated with adverse cardiovascular outcomes. In patients with established coronary artery disease, elevated ImP predicted cardiometabolic deterioration independently of LDL-cholesterol, hs-CRP, and other established biomarkers.^78^ Beyond coronary disease, ImP has been associated with incident heart failure and all-cause mortality in a large multicenter cohort, suggesting broader prognostic relevance across cardiometabolic phenotypes.^77^

These clinical associations align with mechanistic data demonstrating that ImP accelerates atherosclerosis and induces endothelial dysfunction through defined receptor-mediated pathways,^16^ ^,^ ^36^ supporting its role as both a biomarker and a modifiable contributor to cardiovascular risk.

Interventional evidence directly targeting microbial metabolic pathways remains limited. Short-term administration of minimally absorbed antibiotics such as rifaximin suppresses postprandial TMAO production following phosphatidylcholine challenge in humans.^96^ In experimental models, dietary strategies and small-molecule inhibitors of microbial TMA lyase (e.g., 3,3-dimethyl-1-butanol) reduce circulating TMAO, inflammasome activation, and vascular remodeling.^41^ ^,^ ^97^ However, durable, ecosystem-preserving interventions with demonstrated cardiovascular benefit in humans are lacking.

### 4.3. Multi-omic integration of microbial, metabolic, and vascular phenotypes

Integrated metagenomic, metabolomic, and proteomic analyses increasingly support mechanistic links between microbial metabolism and vascular inflammation (Table 5). Population-based studies show that reduced gut microbial gene richness correlates with elevated hs-CRP and other inflammatory markers, linking microbial diversity to systemic inflammation and cardiovascular risk.^98^

**Table 5.** Multi-omic layers linking the gut microbiome to atherosclerosis: representative pathways and vascular implications.

Omic layer	Key findings	Representative molecules/pathways	Vascular implications	References
Metagenomics	Enrichment of microbial genes encoding pathways involved in choline–TMA metabolism and histidine/urocanate pathways	cutC/D (TMA lyases), histidine fermentation genes	Increased microbial capacity to generate atherogenic metabolites (TMAO and ImP)	[16,19,37]
Metabolomics	Circulating microbiome-derived metabolites associated with vascular disease	TMAO, PAGln, ImP	Endothelial dysfunction, platelet activation, inflammatory signaling, plaque progression	[14–17]
Proteomic host response	Coordinated host inflammatory and vascular response pathways associated with microbiome-derived metabolites	Complement proteins (C3, C5), coagulation factors (fibrinogen, D-dimer), endothelial activation markers (VCAM-1, ICAM-1), inflammatory mediators (IL-6, TNF-*α*, CRP)	Systemic inflammation, endothelial dysfunction, thrombosis, plaque progression	[20,99–101]
Integrated multi-omic networks	Integration of metagenomic, metabolomic, and proteomic data reveals coherent microbe → metabolite → host protein networks	Microbial genes linked to circulating metabolites and host inflammatory protein modules	Identification of mechanistic pathways and potential therapeutic targets in atherosclerosis	[8,85,86,102]

Abbreviations: TMA, trimethylamine; TMAO, trimethylamine *N*-oxide; PAGln, phenylacetylglutamine; ImP, imidazole propionate; VCAM-1, vascular cell adhesion molecule 1; ICAM-1, intercellular adhesion molecule 1; IL-6, interleukin 6; TNF-*α*, tumor necrosis factor alpha; CRP, C-reactive protein; C3, complement component 3; C5, complement component 5.

Microbial gene-level analyses reveal that enrichment of TMA-producing genes (cutC/D) and histidine/urocanate metabolism pathways (hutH/urdA) correlates with circulating TMAO and ImP levels, respectively, and with inflammatory proteomic signatures associated with atherosclerosis.^11^ ^,^ ^16^ ^,^ ^37^ Network-based analyses suggest that metabolite-centered modules containing TMAO, PAGln, or ImP mediate part of the association between microbial composition and vascular phenotypes, although direct mediation estimates remain limited.^24^ ^,^ ^103^

Plasma proteomic studies further support these links. In large human cohorts, inflammatory and endothelial activation protein clusters correlate with both atherosclerotic burden and specific microbial taxa or metabolites, reinforcing a mechanistic chain from gut microbial function to host proteomic remodeling and vascular disease.^20^ ^,^ ^21^

### 4.4. Remaining gaps and limitations

Despite substantial progress, several limitations remain. Methodological heterogeneity in dietary assessment, stool sampling, sequencing platforms, and metabolomic quantification complicates cross-study comparisons. Most human data are observational, and although Mendelian randomization and longitudinal analyses strengthen causal inference, residual confounding and pleiotropy cannot be excluded. Non-bacterial components of the gut microbiome—including fungi, viruses, and archaea—remain underexplored, despite their potential immunomodulatory roles.^104^

Critically, randomized controlled trials directly targeting microbial metabolites with cardiovascular endpoints are lacking. Longitudinal studies integrating standardized microbiome profiling, metabolomics, proteomics, and vascular imaging will be essential to establish causality and guide translation.

## 5. People with HIV: a model of gut injury and accelerated atherosclerosis

People with HIV (PWH) represent a uniquely informative human model in which gut barrier disruption, microbial dysbiosis, and chronic immune activation converge to accelerate atherosclerosis. Importantly, this population can be conceptualized as a “human stress-test” of the gut–artery axis, in which key mechanisms described in the general population are amplified, sustained, and more readily detectable in vivo. Despite effective antiretroviral therapy (ART) and durable viral suppression, PWH experience a persistently elevated incidence of myocardial infarction, stroke, and subclinical atherosclerosis, highlighting mechanisms that extend beyond traditional cardiovascular risk factors alone.

### 5.1. Cardiovascular risk in the era of effective ART

Large observational cohorts consistently report a 1.5–2-fold higher risk of cardiovascular events in PWH compared with HIV-negative individuals, even after adjustment for age, smoking, lipid levels, and hypertension.^28^ ^,^ ^29^ ^,^ ^105^ This excess risk persists in virally suppressed individuals and reflects the combined effects of residual immune activation, chronic inflammation, ART-associated metabolic perturbations, and sustained gut mucosal injury.

Inflammatory biomarkers strongly predict cardiovascular outcomes in treated HIV. Elevated circulating IL-6, D-dimer, and markers of monocyte activation remain powerful predictors of morbidity and mortality, underscoring the central role of immune dysregulation in HIV-associated atherogenesis.^106^ ^,^ ^107^ These pathways closely mirror the gut–immune–vascular mechanisms described in non-HIV populations, but are quantitatively amplified and temporally sustained, reinforcing their causal relevance.

### 5.2. Intestinal mucosal damage and dysbiosis in people with HIV

**Figure 3.**
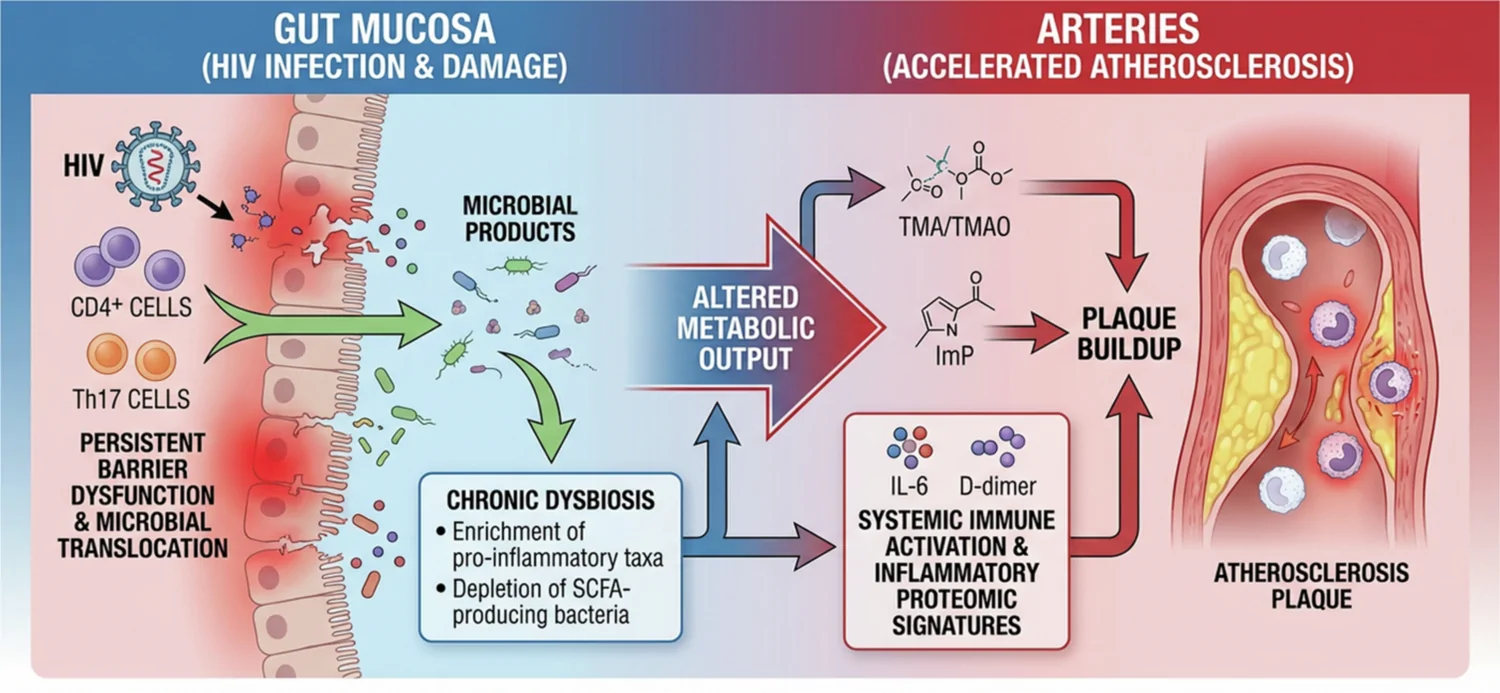
HIV as a model of an amplified gut–artery axis. Gut microbiome–vascular interactions in people living with HIV. HIV infection induces early and sustained gut mucosal damage with depletion of CD4⁺ and Th17 cells, leading to persistent barrier dysfunction and microbial translocation despite effective antiretroviral therapy. Chronic dysbiosis, characterized by enrichment of pro-inflammatory taxa and depletion of SCFA-producing bacteria, sustains systemic immune activation and inflammatory proteomic signatures (e.g., IL-6, D-dimer). Altered microbial metabolic output—including perturbations in TMA/TMAO and emerging evidence for elevated ImP—contributes to accelerated subclinical and clinical atherosclerosis in people living with HIV, illustrating an amplified gut–artery axis. Abbreviations: HIV, Human immunodeficiency virus; CD4⁺, Cluster of differentiation 4–positive T cells; Th17, T helper 17 cells; SCFA, Short-chain fatty acid; IL-6, Interleukin-6; TMA, Trimethylamine; TMAO, Trimethylamine *N*-oxide; ImP, Imidazole propionate.

HIV infection causes early and profound depletion of CD4⁺ T cells within the gut-associated lymphoid tissue, particularly Th17 cells that are critical for epithelial barrier maintenance.^26^ This early mucosal injury is only partially reversible with ART, resulting in persistent epithelial dysfunction and increased intestinal permeability (Figure 3).

As a consequence, microbial translocation persists in treated HIV, evidenced by elevated plasma levels of LPS, soluble CD14 (sCD14), and intestinal fatty acid–binding protein (IFABP).^25^ ^,^ ^27^ These markers correlate with systemic immune activation and predict cardiovascular events, linking gut barrier failure to vascular disease.

Gut microbial dysbiosis in PWH is well characterized and reproducible across cohorts. Typical features include reduced *α*-diversity, enrichment of *Proteobacteria* (notably *Enterobacteriaceae*), and increased abundance of *Prevotella*, *Streptococcus*, and *Erysipelotrichaceae*, alongside depletion of butyrate-producing genera such as *Faecalibacterium* and *Roseburia.* ^108–110^ ART partially reshapes the microbiome but rarely restores a eubiotic state, even after prolonged viral suppression.^111^

Notably, these microbial patterns closely resemble those observed in metabolic syndrome and atherosclerosis in HIV-negative populations, supporting the concept that HIV does not introduce entirely distinct pathways but rather accentuates shared microbiome-mediated mechanisms of cardiometabolic disease.

### 5.3. Gut microbiome–atherosclerosis associations in people with HIV

Emerging longitudinal studies directly link gut microbial features to vascular disease progression in HIV. In a prospective cohort of virally suppressed PWH on ART, enrichment of pro-inflammatory genera (including *Bilophila* and *Ruminococcus 2*), predicted progression of cIMT, independent of traditional cardiovascular risk factors and inflammatory biomarkers^30^ (Table 4).

Microbial signatures associated with metabolic dysregulation also correlate with vascular outcomes. In the same cohort, individuals experiencing significant ART-associated weight gain—particularly those receiving integrase strand transfer inhibitors—showed enrichment of taxa such as *Tyzzerella_4* and *Lactobacillus*, organisms previously linked to adverse metabolic phenotypes.^92^

Collectively, these findings support a contribution of persistent dysbiosis to vascular injury in treated HIV through mechanisms discussed in previous sections (Figure 3). This reinforces the broader concept that microbiome–atherosclerosis associations observed in the general population are recapitulated—and strengthened—in the HIV context.

### 5.4. Microbial metabolites and vascular disease in people with HIV

Alterations in microbial metabolic output accompany dysbiosis in PWH (Table 4). Several studies have examined the choline–TMA–TMAO pathway in HIV, with heterogeneous results. Plasma TMA concentrations are frequently elevated in PWH, whereas TMAO levels vary depending on ART exposure, renal function, immune activation, and microbial composition.^89^ ^,^ ^91^ ^,^ ^112^ ^,^ ^113^

In one well-characterized cohort assessed with CCTA, TMA—but not TMAO—was independently associated with CAC score, number of calcified plaque segments, and total plaque volume, even after multivariable adjustment for traditional cardiovascular risk factors.^89^ These findings suggest that TMA may serve as a more proximal marker of microbial activity and vascular burden in HIV than TMAO.

Prospective data indicate that TMAO may nevertheless contribute to vascular progression in specific contexts. In two longitudinal HIV cohorts, higher baseline TMAO predicted incident carotid plaque progression by ultrasound, implicating microbial choline metabolism in HIV-associated vascular injury.^90^

More recently, ImP has emerged as a candidate metabolite of interest in HIV. Elevated circulating ImP has been reported in PWH with obstructive coronary artery disease and a dysbiotic microbiome enriched for *Ruminococcus gnavus* and *Veillonella.* ^93^ Several taxa implicated in ImP biosynthesis—including *R. gnavus*, *Veillonella*, *Clostridium* spp., *Eggerthella*, and UrdA-positive *Streptococcus*—^19^ ^,^ ^114^ ^,^ ^115^ are enriched in HIV-associated dysbiosis. These observations suggest that HIV may represent a clinical setting in which metabolite-driven mechanisms can be interrogated with enhanced signal-to-noise relative to the general population. Given ImP’s proposed causal role in atherosclerosis in the general population, its contribution to vascular disease in HIV warrants focused mechanistic and longitudinal investigation.

### 5.5. Proteomic and inflammatory correlates

High-throughput proteomic studies in treated PWH consistently demonstrate persistent upregulation of inflammatory, endothelial activation, and coagulation-related proteins despite viral suppression. In a large proximity-extension-assay study profiling approximately 1,472 plasma proteins, multiple differentially expressed proteins in PWH mapped to innate immune activation, lipid metabolism, and endothelial function, and a subset predicted incident cardiovascular disease.^99^ Importantly, several of these proteins correlated with markers of microbial translocation (e.g., IFABP, sCD14) and with the abundance of specific gut bacterial taxa, supporting a mechanistic axis linking gut injury to systemic proteomic remodeling and vascular risk. These proteomic signatures overlap substantially with inflammatory pathways implicated in atherosclerosis in HIV-negative populations, further reinforcing the shared—and amplified—nature of these mechanisms.

Targeted biomarker studies corroborate these findings. Persistently elevated IL-6, D-dimer, and endothelial activation markers predict cardiovascular events in treated HIV, emphasizing the clinical relevance of chronic immune activation.^106^ ^,^ ^107^

### 5.6. Pathogenic model and translational implications

Collectively, evidence supports a multifactorial model of accelerated atherosclerosis in HIV in which (Figure 3): (i) early and persistent gut mucosal injury permits ongoing microbial translocation; (ii) chronic microbial-driven immune activation produces an adverse circulating cytokine and proteomic milieu; and (iii) altered microbial metabolic output—including TMA/TMAO and potentially ImP—provides additional pro-atherogenic signals acting on endothelial cells, immune cells, and platelets.

Viewed through this framework, HIV serves not only as a distinct clinical population but also as a mechanistically informative model that strengthens causal inference for the gut–artery axis by amplifying otherwise subtler pathways observed in the general population.

Although the magnitude and persistence of gut barrier dysfunction and immune activation are amplified in HIV, the underlying pathways are not HIV-specific and substantially overlap with those operating in the broader population. Findings derived from people with HIV should therefore be regarded as mechanistically informative and hypothesis-generating for non-HIV populations, while requiring direct validation before broader clinical generalization.

These convergent pathways plausibly explain residual cardiovascular risk in PWH despite viral suppression and aggressive management of traditional risk factors.

From a translational perspective, HIV offers a tractable human context in which microbiome-targeted strategies may yield disproportionate benefit. Potential approaches include reduction of pro-atherogenic microbial metabolites, restoration of SCFA-producing communities, and targeted modulation of host inflammatory pathways. Emerging evidence identifying the ImP–imidazoline-1 receptor axis as a modifiable driver of atherosclerosis further expands the therapeutic landscape, although dedicated studies in HIV are required.

## 6. Beyond the microbiome: metabolomics and proteomics as integrative layers

The mechanisms linking gut microbial dysbiosis to atherosclerosis described above converge at the level of microbial metabolic output and host biological response. While individual metabolites such as TMAO, PAGln, and ImP provide mechanistic entry points into the gut–artery axis, their vascular relevance ultimately depends on how these signals are integrated within host metabolic, inflammatory, and endothelial pathways.

Metabolomics and proteomics offer complementary and functionally informative layers to capture this integration. Metabolomics reflects the combined activity of microbial and host metabolism, bridging microbial gene content with systemic exposure to bioactive compounds. Proteomics, in turn, delineates coordinated host response pathways—including inflammation, endothelial activation, coagulation, and immune signaling—that translate microbial-derived cues into atherosclerotic phenotypes.

Together, these omic layers enable a systems-level view of microbiome–atherosclerosis interactions, extending the integrated host–microbiome crosstalk outlined earlier. Rather than introducing additional actors, this section illustrates how multi-omic integration clarifies pathway structure, strengthens mechanistic coherence, and prioritizes translational targets within the gut–artery axis.

### 6.1. Metabolomics: chemical mediators of the gut–artery axis

Metabolomics provides quantitative profiling of small molecules in biofluids and tissues, capturing the integrated metabolic state of the host and its microbiota.^24^ In microbiome–atherosclerosis research, metabolomics is particularly valuable as an intermediate layer that connects microbial genetic potential to vascular phenotypes by identifying candidate chemical mediators that can be mechanistically tested and, in some cases, translated into clinically scalable biomarkers.

Discovery metabolomics has been instrumental in identifying microbiome-linked metabolites that were subsequently mapped to defined biological pathways. Canonical examples include TMAO,^14^ ^,^ ^17^ PAGln,^15^ and ImP.^16^ ^,^ ^19^ Together, these discoveries illustrate a central principle: metabolomics prioritizes biologically plausible mediators, but causal inference requires targeted experimental validation (see Section 3 for mechanistic effects).

These metabolites exemplify distinct but convergent biochemical routes: TMAO from quaternary amines, PAGln from aromatic amino acids, and ImP from histidine fermentation. Together, they form a conceptual framework linking dietary substrates, microbial metabolic pathways, and vascular pathology.

### 6.2. Proteomics: systemic fingerprints of gut-driven inflammation

Proteomics complements metabolomics by capturing downstream host responses to microbial products and metabolites. High-throughput plasma proteomic platforms have identified reproducible signatures of inflammation, endothelial activation, coagulation, and complement activation in atherosclerosis.^20^ ^,^ ^100^ ^,^ ^101^ Plasma proteomic pathways linked to microbiome-driven inflammation in atherosclerosis are shown in Table 5.

Importantly, many proteins associated with vascular disease also correlate with microbial features or circulating microbial metabolites, supporting a microbe → metabolite → host protein axis. Circulating protein clusters enriched for IL-6 signaling, acute-phase responses, and endothelial adhesion molecules correlate with TMAO and ImP levels and associate with plaque burden and instability.^16^ ^,^ ^21^ These associations provide systems-level evidence that microbial metabolism propagates into coordinated host inflammatory networks relevant to atherogenesis.

### 6.3. Multi-omic integration: systems biology of microbiome–atherosclerosis interactions

Joint analysis of metagenomics, metabolomics, and proteomics enables empirical mapping of links between microbial functions, circulating metabolites, and host response pathways (Table 5). Integration is most informative when it supports a directional biological framework—microbial genes and pathways → metabolites → host effector networks—thereby prioritizing testable hypotheses and translational targets.

Population-scale studies demonstrate that microbial composition explains a substantial proportion of metabolomic variance, reinforcing metabolites as intermediate phenotypes that reduce dimensionality and enhance biological interpretability.^8^ ^,^ ^102^ In cardiovascular research, microbial gene-level features can anchor metabolite signals to specific enzymatic capacities, such as choline-to-TMA conversion or histidine/urocanate metabolism, which can then be linked to host proteomic modules to identify candidate receptors and downstream effector pathways.^16^ ^,^ ^19^ ^,^ ^37^ ^,^ ^116^

Recent multi-omic meta-analyses integrating microbiome and host layers have identified reproducible microbe–metabolite–host feature sets associated with cardiometabolic and vascular phenotypes.^85^ ^,^ ^86^ Genetically informed and mediation-oriented analyses further prioritize metabolites that lie on putative causal paths between upstream exposures and downstream vascular risk, while highlighting where residual confounding or pleiotropy may persist.^86^ Practical considerations related to analytical harmonization, data integration, and reproducibility^117^ are discussed in Section 8.

Together, these layered datasets demonstrate how multi-omic integration can move beyond correlation toward mechanistic prioritization, identifying specific microbial enzymes, metabolites, host responses, and atherosclerotic phenotypes, and are essential for advancing microbiome research toward clinical translation. However, translation of multi-omic signatures into routine clinical practice remains limited by differences in sample handling, analytical platforms, feature annotation, normalization, and data-integration pipelines. For metabolomics in particular, platform-dependent metabolite coverage, incomplete harmonization of reference standards, and inter-laboratory variability complicate cross-study comparison. Standardized workflows and external validation in independent cohorts will therefore be required before multi-omic signatures can reliably support clinical decision-making.

## 7. Clinical translation: biomarkers and therapeutic opportunities

Insights into microbiome–atherosclerosis interactions have substantial translational implications. Gut microbiota–derived metabolites provide reproducible, quantifiable readouts of microbial activity that integrate diet, microbial function, and host metabolism. In parallel, mechanistic studies identifying specific microbial enzymes and host receptors have opened new avenues for targeted intervention. Together, these advances position the gut–artery axis as a promising—though still evolving—frontier for precision cardiovascular medicine.

### 7.1. Circulating microbial metabolites as biomarkers

Targeted metabolomic assays allow reliable quantification of gut-derived metabolites in plasma and have been applied across large cohorts. Among these, TMAO, PAGln, and ImP represent prototypical examples with robust epidemiologic and mechanistic support.

Elevated TMAO consistently predicts adverse cardiovascular outcomes, although its interpretation is modulated by renal function, diet, and host FMO3 activity.^17^ ^,^ ^42^ PAGln is primarily linked to thrombotic risk via adrenergic signaling and predicts cardiovascular events independently of lipid markers.^15^ ImP has emerged as a particularly informative marker of metabolically active atherosclerosis, associating with plaque inflammation and adverse cardiometabolic outcomes beyond traditional risk factors, supported by a defined receptor–signaling axis.^16^ ^,^ ^19^ ^,^ ^78^

Beyond single metabolites, stool-based metagenomic markers—such as microbial gene abundance encoding choline–TMA lyases (cutC/D) or histidine-to-ImP biosynthetic pathways (hutH/urdA)—offer non-invasive measures of microbial metabolic potential and correlate with circulating metabolite levels in integrated datasets.^19^ ^,^ ^74^ Composite signatures combining metabolites with inflammatory or proteomic markers may further enhance risk stratification, although large prospective validation studies remain necessary.

From a translational perspective, these metabolites occupy different positions along the clinical-development continuum. TMAO currently has the greatest maturity as a prognostic cardiovascular biomarker, although its specificity is limited by the influence of renal function, diet, and host metabolism. PAGln represents an emerging marker of platelet-mediated thrombotic risk and a potentially targetable adrenergic-signaling pathway but requires broader external validation. ImP remains primarily a mechanistically informative candidate mediator, with promising biomarker and therapeutic potential that awaits longitudinal and interventional confirmation.

### 7.2. Therapeutic modulation of the gut–artery axis

**Figure 4.**
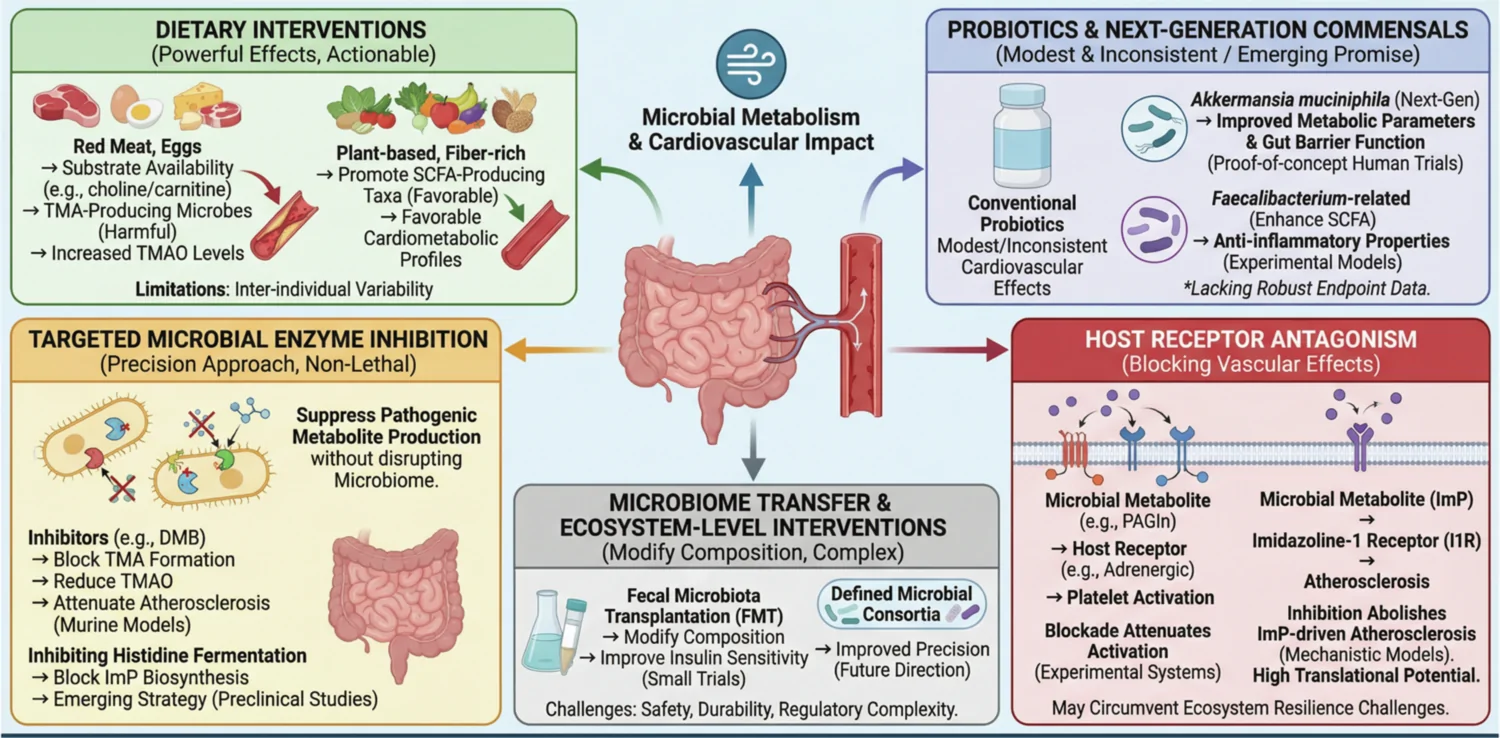
Translational opportunities targeting the gut–artery axis. Translational strategies targeting microbiome-driven atherosclerosis. Potential intervention points along the gut–artery axis include dietary modulation to reduce substrate availability for pro-atherogenic microbial pathways, restoration of SCFA-producing communities, targeted inhibition of microbial enzymes (e.g., TMA lyase), and blockade of host receptors mediating metabolite effects (e.g., adrenergic receptors for PAGln, imidazoline-1 receptor for ImP). Circulating microbial metabolites and microbial gene signatures offer complementary biomarker strategies for risk stratification and treatment monitoring. Abbreviations: SCFA, Short-chain fatty acid; TMA, Trimethylamine; PAGln, Phenylacetylglutamine; ImP, Imidazole propionate; DMB, 3,3-dimethyl-1-butanol.

Several complementary strategies are being explored to attenuate the cardiovascular impact of harmful microbial metabolism or to restore protective microbial functions (Table 6, Figure 4). Evidence supporting each approach varies substantially in maturity.

**Table 6.** Microbiome-targeted strategies with potential to modulate atherosclerosis.

Strategy	Target/mechanism	Potential vascular effects	Representative references
Dietary modulation (e.g., reduced choline/carnitine intake, increased fiber)	Alters microbial substrate availability and promotes SCFA-associated microbial communities	Reduced generation of pro-atherogenic metabolites such as TMAOand favorable shifts in microbial and cardiometabolic profiles	[14,17,22,118,119]
Probiotics/prebiotics	Enrichment of beneficial bacteria and restoration of microbial metabolic balance	Improved endothelial function and inflammatory biomarkers; favorable metabolic and gut-barrier effects in early studies	[120,121,123,124]
Microbial enzyme inhibition	Target microbial enzymes such as cutC/D responsible for TMA production	Reduced TMA/TMAO generation and attenuated atherosclerosis; potential reduction in thrombogenic signaling	[41,116]
Host receptor antagonism	Blockade of host receptors mediating microbial metabolite effects (e.g., adrenergic receptors for PAGln; I1R/nischarin for ImP)	Reduced metabolite-driven platelet activation and/or atherogenic inflammatory signali ng	[15,16]
Fecal microbiota transplantation	Ecosystem-level replacement of dysbiotic microbiota	Improved insulin sensitivity and metabolic profile; cardiovascular benefit remains unproven	[122]
Multi-omics–guided target discovery/precision microbiome approaches	Integration of microbial genes, metabolites, and host pathways to identify candidate intervention targets	Prioritization of pathway-specific therapeutic targets	[85,86]

Abbreviations: TMA, trimethylamine; TMAO, trimethylamine *N*-oxide; cutC/D, choline trimethylamine-lyase gene cluster; FMT, fecal microbiota transplantation.

#### 7.2.1. Dietary interventions

Dietary patterns exert powerful effects on gut microbial composition and metabolic output. Diets rich in red meat and eggs increase substrate availability for TMA-producing microbes and raise circulating TMAO levels, whereas plant-based, fiber-rich diets promote SCFA–producing taxa and favorable cardiometabolic profiles.^14^ ^,^ ^17^ ^,^ ^22^ ^,^ ^118^ Dietary modification therefore remains the most immediately actionable strategy to influence microbial metabolism, although inter-individual variability in microbial response limits uniform efficacy.^119^

#### 7.2.2. Probiotics and next-generation commensals

Evidence for conventional probiotics remains limited and heterogeneous. In contrast, next-generation commensals such as *Akkermansia muciniphila* have demonstrated improvements in metabolic parameters and gut barrier function in proof-of-concept human trials.^123^ Commensals that enhance SCFA production, including *Faecalibacterium*-related taxa, exhibit anti-inflammatory properties in experimental models,^124^ but robust cardiovascular endpoint data are lacking.

#### 7.2.3. Targeted microbial enzyme inhibition

Small-molecule inhibition of microbial enzymes represents a precision approach to suppress pathogenic metabolite production without broadly disrupting the microbiome. Non-lethal inhibitors of microbial TMA formation, such as 3,3-dimethyl-1-butanol (DMB), reduce circulating TMAO levels and attenuate atherosclerosis in murine models.^41^ Comparable strategies aimed at reducing microbial ImP production represent a potential future approach, although current experimental evidence more directly supports targeting the downstream ImP–imidazoline-1 receptor axis.^16^

#### 7.2.4. Host receptor antagonism

An alternative strategy is to block host receptors that mediate the vascular effects of microbial metabolites. PAGln-driven platelet activation is attenuated by adrenergic receptor blockade in experimental systems.^15^ More strikingly, inhibition of the ImP–imidazoline-1 receptor axis abolishes ImP-driven atherosclerosis in mechanistic models, identifying a tractable host target with high translational potential.^16^ Such approaches may circumvent challenges related to microbial ecosystem resilience.

#### 7.2.5. Microbiome transfer and ecosystem-level interventions

Fecal microbiota transplantation (FMT) can modify microbial composition and improve insulin sensitivity in small, randomized trials.^122^ However, safety concerns, durability of effects, and regulatory complexity currently limit its application for cardiovascular prevention. More refined ecosystem-level approaches, including defined microbial consortia, may offer improved precision in the future.

Several microbiome-targeted approaches are now progressing toward early clinical evaluation, including precision dietary interventions, next-generation probiotics or defined microbial consortia, and pathway-specific strategies such as microbial enzyme inhibition. However, most cardiovascular evidence remains preclinical or is limited to intermediate metabolic and inflammatory outcomes, underscoring the need for controlled studies incorporating vascular and clinical endpoints.

### 7.3. Challenges for clinical implementation

Despite encouraging mechanistic and translational data, several challenges must be addressed before microbiome-informed strategies can be implemented clinically. Microbial metabolite levels are influenced by diet, renal function, medications, and host genetics, complicating threshold-based interpretation. Long-term safety of sustained microbial or metabolic manipulation remains uncertain, particularly in populations with multimorbidity or polypharmacy, such as people living with HIV.

Critically, randomized controlled trials targeting microbial metabolites with cardiovascular endpoints are lacking. Future trials should incorporate mechanistic readouts—including microbiome functional profiling, metabolomics, proteomics, and vascular imaging—to establish causality, identify responders, and detect off-target effects. In parallel, greater standardization of sampling procedures, sequencing methodologies, and analytical pipelines will be essential to improve reproducibility and facilitate cross-cohort validation of microbiome-derived biomarkers.

## 8. Standards, pitfalls, and reproducibility in microbiome–cardiovascular research

As microbiome research moves toward clinical translation, rigor, reproducibility, and transparency are paramount. Studies linking gut microbial features to atherosclerosis face unique methodological challenges, spanning biological variability, technical heterogeneity, and analytical complexity. Addressing these challenges is essential to ensure that mechanistic insights and translational claims are robust, generalizable, and clinically meaningful.

### 8.1. Methodological heterogeneity and standardization

Human microbiome studies are particularly sensitive to technical variation. Early assessments by the Microbiome Quality Control (MBQC) consortium demonstrated that differences in DNA extraction methods, primer choice, sequencing platforms, and bioinformatic pipelines can introduce variability comparable in magnitude to true biological differences.^125^ ^,^ ^126^ These effects are especially consequential in cardiovascular research, where effect sizes are often modest and confounding is substantial.

To mitigate these issues, standardized protocols and transparent reporting are essential. The STORMS (Strengthening The Organization and Reporting of Microbiome Studies) checklist provides a community-endorsed framework for reporting microbiome studies and specifies essential metadata, including sample collection and storage conditions, DNA extraction methods, sequencing chemistry, bioinformatic workflows, and statistical approaches.^127^ Adoption of such standards facilitates cross-study comparison, replication, and meta-analysis.

In cardiovascular microbiome studies, additional metadata are critical. Detailed dietary assessment, medication use (particularly antibiotics, statins, metformin, and proton pump inhibitors), renal function, and inflammatory status should be systematically recorded, as each strongly influences microbial composition and circulating metabolite levels. Failure to account for these factors can lead to spurious associations or obscure true biological signals.

Table 7 summarizes the main methodological sources of variability in the microbiome analysis.

**Table 7.** Methodological sources of variability in microbiome research.

Source of variability	Impact on findings	Mitigation strategies
DNA extraction and sequencing protocols	Systematic bias in taxonomic and functional profiles	Use of standardized, benchmarked protocols and transparent reporting (e.g., MBQC guidelines)
Diet and medication exposure	Confounding of microbial composition and metabolite levels	Structured dietary assessment and comprehensive medication metadata
Inter-individual biological variability	Reduced signal-to-noise ratio in cross-sectional analyses	Longitudinal study designs with repeated sampling
Asynchronous or compartment-specific omic sampling	Misalignment between microbial features and host responses	Harmonized, time-matched multi-omic collection workflows

Abbreviations: MBQC, Microbiome quality control project; omic, high-throughput molecular profiling.

### 8.2. Study design considerations and causal inference

Most human studies linking the gut microbiome to atherosclerosis are cross-sectional, limiting inference about temporality and causality. Longitudinal designs with repeated microbiome sampling and concurrent vascular imaging are better suited to disentangle cause from consequence but remain relatively scarce.

Mendelian randomization has emerged as a valuable tool to strengthen causal inference by leveraging host genetic variants associated with microbial taxa or metabolites.^38^ ^,^ ^39^ However, such analyses rely on strong assumptions and are vulnerable to horizontal pleiotropy and weak instrument bias. Results should therefore be interpreted as supportive rather than definitive evidence of causality.

Experimental models remain indispensable. Germ-free and gnotobiotic mice, fecal microbiota transplantation, and targeted manipulation of microbial metabolic pathways have provided critical causal evidence—particularly for TMAO and ImP. Nevertheless, differences between murine and human physiology, diet, and microbiome composition limit direct extrapolation. Integration of human observational data with mechanistic experimentation remains the most robust approach.

### 8.3. Metabolomics and proteomics: analytical considerations

Metabolomic and proteomic measurements introduce additional layers of complexity. Targeted mass-spectrometry assays offer high sensitivity and reproducibility for specific metabolites such as TMAO, PAGln, and ImP, whereas untargeted platforms provide broader coverage at the cost of increased analytical variability.^24^ Harmonization of analytical pipelines, use of internal standards, and cross-laboratory validation are essential for reproducibility.

Proteomic platforms similarly vary in depth and sensitivity. Proximity-extension assays enable high-throughput profiling of inflammatory and vascular proteins but are biased toward predefined panels, whereas mass-spectrometry–based proteomics offers unbiased discovery with lower throughput. Transparent reporting of platform choice, normalization procedures, and batch-correction strategies is critical, particularly when integrating datasets across studies.

### 8.4. Data integration, open science, and FAIR principles

Multi-omic integration requires high-quality, interoperable datasets. The FAIR principles—Findable, Accessible, Interoperable, and Reusable—provide a foundation for responsible data stewardship.^117^ Public deposition of raw sequencing data, metabolomic spectra, proteomic measurements, and associated metadata in repositories such as NCBI SRA, MetaboLights, and PRIDE enables independent validation and secondary analyses.

Population-scale studies illustrate the importance of extensive phenotyping and harmonized metadata to disentangle host, environmental, and technical drivers of microbiome variation.^119^ Network-based and mediation analyses can help prioritize mechanistic pathways, but results depend critically on data quality and completeness.

Importantly, negative and null findings should be reported to reduce publication bias and prevent overinterpretation of weak or cohort-specific associations.

### 8.5. Translational caution and ethical considerations

While enthusiasm for microbiome-based interventions is high, premature clinical implementation carries risks. The gut microbiome is a complex, adaptive ecosystem, and sustained perturbation may have unintended consequences. Long-term safety data for targeted microbial enzyme inhibitors, receptor antagonists, or live biotherapeutics are limited, particularly in vulnerable populations with multimorbidity or immune dysregulation.

Ethical considerations include equitable access to microbiome-based diagnostics and therapies, transparency regarding uncertainty, and avoidance of exaggerated claims. In cardiovascular prevention, where effective standard-of-care therapies already exist, microbiome-targeted approaches should be positioned as complementary rather than replacement strategies until robust outcome data are available.

Progress in microbiome–atherosclerosis research depends on rigorous methodology, standardized reporting, and careful causal inference. As the field advances, disciplined interpretation and transparent reporting will be essential to translate microbiome science into clinically meaningful cardiovascular insight.

## 9. Conclusions and future perspectives

Over the past decade, the gut microbiome has emerged from a correlative curiosity to a mechanistically grounded contributor to atherosclerotic cardiovascular disease. Accumulating evidence now supports a model in which gut microbial communities influence vascular pathology through coordinated effects on intestinal barrier integrity, immune activation, and—most decisively—the production of bioactive metabolites that directly modulate endothelial, immune, and platelet function. These processes form an integrated gut–artery axis that helps explain residual cardiovascular risk beyond traditional factors.

Among microbiome-derived mediators, microbial metabolites have provided the most compelling mechanistic links. Trimethylamine *N*-oxide and phenylacetylglutamine exemplify how diet–microbe interactions translate into atherothrombotic signaling through effects on lipid handling and platelet reactivity. More recently, imidazole propionate has fundamentally advanced the field by establishing a putative causal, receptor-mediated pathway linking microbial histidine metabolism to endothelial dysfunction, inflammatory activation, and plaque development. The identification of the ImP–imidazoline-1 receptor–mTORC1 axis represents a conceptual shift: from microbial metabolites as associative biomarkers to defined molecular effectors of vascular disease.

Multi-omic approaches have been critical in enabling this transition. Integration of metagenomics, metabolomics, and proteomics has revealed coherent microbial gene–metabolite–host protein networks associated with subclinical and clinical atherosclerosis, strengthening causal inference and prioritizing specific enzymes, pathways, and receptors for intervention. These systems-level insights underscore that microbial composition alone is insufficient to explain cardiovascular risk; functional output and host response are the decisive determinants.

People with HIV provide a powerful human model in which these mechanisms are amplified. Persistent gut barrier disruption, dysbiosis, and immune activation despite effective antiretroviral therapy recapitulate—and intensify—the gut–vascular pathways observed in the general population. In this context, microbial metabolites and proteomic signatures offer both mechanistic insight and translational opportunity, highlighting how microbiome-driven processes may contribute to accelerated vascular aging in chronic inflammatory states.

Looking forward, several priorities will shape the next phase of microbiome–atherosclerosis research. First, longitudinal human studies with repeated microbiome, metabolomic, proteomic, and vascular imaging assessments are essential to establish temporality and refine causal models. Second, interventional trials targeting microbial metabolic pathways—whether through diet, enzyme inhibition, or host receptor antagonism—must incorporate mechanistic endpoints to confirm on-target effects and identify responders. Third, greater attention to methodological standardization, data sharing, and reproducibility is required to ensure that findings are robust and generalizable across populations. Together, these findings support a shift from descriptive microbiome–atherosclerosis associations toward a metabolite-centered, mechanism-driven framework that enables causal inference, biomarker prioritization, and therapeutic targeting.

From a translational perspective, microbiome-informed cardiovascular medicine is unlikely to replace established therapies in the near term. Instead, its greatest potential lies in complementing existing risk stratification and prevention strategies by identifying biologically distinct pathways of residual risk and enabling more precise, mechanism-based interventions. As demonstrated by recent advances surrounding imidazole propionate, the field is now poised to move from descriptive associations toward actionable molecular targets.

In summary, the gut microbiome represents a dynamic and modifiable contributor to atherosclerosis, with microbial metabolites emerging as key mechanistic mediators linking gut ecology to vascular disease. Continued integration of mechanistic experimentation with rigorous human multi-omic studies will be essential to translate this metabolite-centered biology into meaningful clinical benefit.

## Data Availability

Data sharing not applicable—no new data generated.

## Language editing assistance

Language editing assistance was provided using AI-based tools, including ChatGPT 5.2 (OpenAI) and Paperpal. These tools were used to improve clarity, grammar, and fluency. The authors are solely responsible for the final content and interpretation.

## Figure preparation assistance

Figures were created with the assistance of AI-based design software (Oreate AI). The tool was used for graphical layout and visualization only. All scientific content, data interpretation, and final figure selection were performed and verified by the authors.

## References

[1] World Health Organization Cardiovascular diseases (CVDs): fact sheet. 2023. Geneva (Switzerland): World Health Organization. https://www.who.int/news-room/fact-sheets/detail/cardiovascular-diseases-(cvds).

[2] Roth GA , Mensah GA , Johnson CO , Addolorato G , Ammirati E , Baddour LM , Barengo NC , Beaton AZ , Benjamin EJ , Benziger CP , et al. GBD-NHLBI-JACC global burden of cardiovascular diseases writing group. Global burden of cardiovascular diseases and risk factors, 1990–2019: update from the GBD 2019 study. J Am Coll Cardiol. 2020;76(25):2982–3021. doi: 10.1016/j.jacc.2020.11.010. 33309175 PMC7755038

[3] Ridker PM , Everett BM , Thuren T , MacFadyen JG , Chang WH , Ballantyne C , Fonseca F , Nicolau J , Koenig W , Anker SD , et al. CANTOS trial group. Antiinflammatory therapy with canakinumab for atherosclerotic disease. NEJM. 2017;377(12):1119–1131. doi: 10.1056/NEJMoa1707914. 28845751

[4] Libby P . The changing landscape of atherosclerosis. Nature. 2021 Apr;592(7855):524–533. doi: 10.1038/s41586-021-03392-8. 33883728

[5] Tang WH , Hazen SL . The gut microbiome and its role in cardiovascular diseases. Circulation. 2017 Mar 14;135(11):1008–1010. doi: 10.1161/CIRCULATIONAHA.116.024251. 28289004 PMC5354081

[6] Witkowski M , Weeks TL , Hazen SL . Gut microbiota and cardiovascular disease. Circ Res. 2020 Jul 31;127(4):553–570. doi: 10.1161/CIRCRESAHA.120.316242. 32762536 PMC7416843

[7] Hou K , Wu ZX , Chen XY , Wang JQ , Zhang D , Xiao C , Zhu D , Koya JB , Wei L , Li J . Microbiota in health and diseases. Signal Transduct Target Ther. 2022 Apr 23;7(1):135. doi: 10.1038/s41392-022-00974-4. 35461318 PMC9034083

[8] Wikoff WR , Anfora AT , Liu J , Schultz PG , Lesley SA , Peters EC , Siuzdak G . Metabolomics analysis reveals large effects of gut microflora on mammalian blood metabolites. Proc Natl Acad Sci U S A. 2009;106(10):3698–3703. doi: 10.1073/pnas.0812874106. 19234110 PMC2656143

[9] Karlsson FH , Fåk F , Nookaew I , Tremaroli V , Fagerberg B , Petranovic D , Bäckhed F , Nielsen J . Symptomatic atherosclerosis is associated with an altered gut metagenome. Nat Commun. 2012;3:1245. doi: 10.1038/ncomms2266. 23212374 PMC3538954

[10] Jie Z , Xia H , Zhong SL , Feng Q , Li S , Liang S , Liu Z , Gao Y , Zhao H , Zhang D , et al. The gut microbiome in atherosclerotic cardiovascular disease. Nat Commun. 2017 Oct 10;8(1):845. doi: 10.1038/s41467-017-00900-1. 29018189 PMC5635030

[11] Kurilshikov A , van den Munckhof ICL , Chen L , Bonder MJ , Schraa K , Rutten JHW , Riksen NP , de Graaf J , Oosting M , Sanna S , et al. LifeLines DEEP cohort study, BBMRI metabolomics consortium; slagboom PE, xavier RJ, kuipers F, hofker MH, wijmenga C, netea MG, zhernakova A, fu J. Gut microbial associations to plasma metabolites linked to cardiovascular phenotypes and risk. Circ Res. 2019;124(12):1808–1820. doi: 10.1161/CIRCRESAHA.118.314642. 30971183

[12] Fan Y , Pedersen O . Gut microbiota in human metabolic health and disease. Nat Rev Microbiol. 2021 Jan;19(1):55–71. doi: 10.1038/s41579-020-0433-9. 32887946

[13] Haghikia A , Zimmermann F , Schumann P , Jasina A , Roessler J , Schmidt D , Heinze P , Kaisler J , Nageswaran V , Aigner A , et al. Propionate attenuates atherosclerosis by immune-dependent regulation of intestinal cholesterol metabolism. Eur Heart J. 2022;43(6):518–533. doi: 10.1093/eurheartj/ehab644. 34597388 PMC9097250

[14] Wang Z , Klipfell E , Bennett BJ , Koeth R , Levison BS , Dugar B , Feldstein AE , Britt EB , Fu X , Chung Y , et al. Gut flora metabolism of phosphatidylcholine promotes cardiovascular disease. Nature. 2011;472(7341):57–63. doi: 10.1038/nature09922. 21475195 PMC3086762

[15] Nemet I , Saha PP , Gupta N , Zhu W , Romano KA , Skye SM , Cajka T , Mohan ML , Li L , Wu Y , et al. A cardiovascular disease-linked gut microbial metabolite acts via adrenergic receptors. Cell. 2020;180(5):862–877.e22. doi: 10.1016/j.cell.2020.02.016. 32142679 PMC7402401

[16] Mastrangelo A , Robles-Vera I , Mañanes D , Galán M , Femenía-Muiña M , Redondo-Urzainqui A , Barrero-Rodríguez R , Papaioannou E , Amores-Iniesta J , Devesa A , et al. Imidazole propionate is a driver and therapeutic target in atherosclerosis. Nature. 2025;645(8079):254–261. doi: 10.1038/s41586-025-09263-w. 40670786 PMC12408353

[17] Koeth RA , Wang Z , Levison BS , Buffa JA , Org E , Sheehy BT , Britt EB , Fu X , Wu Y , Li L , et al. Intestinal microbiota metabolism of L-carnitine, a nutrient in red meat, promotes atherosclerosis. Nat Med. 2013;19(5):576–585. doi: 10.1038/nm.3145. 23563705 PMC3650111

[18] (a) Chen ML , Zhu XH , Ran L , Lang HD , Yi L , Mi MT . Trimethylamine-N-Oxide induces vascular inflammation by activating the NLRP3 inflammasome through the SIRT3-SOD2-mtROS signaling pathway. J Am Heart Assoc. 2017 Sep 4;6(9), e006347. doi: 10.1161/JAHA.117.006347. 28871042 PMC5634285

[19] Molinaro A , Bel Lassen P , Henricsson M , Wu H , Adriouch S , Belda E , Chakaroun R , Nielsen T , Bergh P , Rouault C , et al. Imidazole propionate is increased in diabetes and associated with dietary patterns and altered microbial ecology. Nat Commun. 2020 Nov 18;11(1):5881. doi: 10.1038/s41467-020-19589-w. 33208748 PMC7676231

[20] Theofilatos K , Stojkovic S , Hasman M , van der Laan SW , Baig F , Barallobre-Barreiro J , Schmidt LE , Yin S , Burnap S , Singh B , et al. Proteomic Atlas of atherosclerosis: the contribution of proteoglycans to sex differences, plaque phenotypes, and outcomes. Circ Res. 2023;133(7):542–558. doi: 10.1161/CIRCRESAHA.123.322590. 37646165 PMC10498884

[21] Wang Z , Peters BA , Bryant M , Hanna DB , Schwartz T , Wang T , Sollecito CC , Usyk M , Grassi E , Wiek F , et al. Gut microbiota, circulating inflammatory markers and metabolites, and carotid artery atherosclerosis in HIV infection. Microbiome. 2023 May 27;11:119. doi: 10.1186/s40168-023-01566-2. 37237391 PMC10224225

[22] De Filippo C , Cavalieri D , Di Paola M , Ramazzotti M , Poullet JB , Massart S , Collini S , Pieraccini G , Lionetti P . Impact of diet in shaping gut microbiota revealed by a comparative study in children from Europe and rural Africa. Proc Natl Acad Sci U S A. 2010 Aug 17;107(33):14691–14696. doi: 10.1073/pnas.1005963107. 20679230 PMC2930426

[23] (a) Qin Y , Havulinna AS , Liu Y , Jousilahti P , Ritchie SC , Tokolyi A , Sanders JG , Valsta L , Brożyńska M , Zhu Q , et al. Combined effects of host genetics and diet on human gut microbiota and incident disease in a single population cohort. Nat Genet. 2022 Feb;54(2):134–142. doi: 10.1038/s41588-021-00991-z. 35115689 PMC9883041

[24] Wishart DS , Guo A , Oler E , Wang F , Anjum A , Peters H , Dizon R , Sayeeda Z , Tian S , Lee BL , et al. HMDB 5.0: the human metabolome database for 2022. Nucleic Acids Res. 2022;50(D1):D622–D631. doi: 10.1093/nar/gkab1062. 34986597 PMC8728138

[25] Brenchley JM , Price DA , Schacker TW , Asher TE , Silvestri G , Rao S , Kazzaz Z , Bornstein E , Lambotte O , Altmann D , et al. Microbial translocation is a cause of systemic immune activation in chronic HIV infection. Nat Med. 2006;12(12):1365–1371. doi: 10.1038/nm1511. 17115046

[26] Brenchley JM , Schacker TW , Ruff LE , Price DA , Taylor JH , Beilman GJ , Nguyen PL , Khoruts A , Larson M , Haase AT , et al. CD4+ T cell depletion during all stages of HIV disease occurs predominantly in the gastrointestinal tract. J Exp Med. 2004 Sep 20;200(6):749–759. doi: 10.1084/jem.20040874. 15365096 PMC2211962

[27] Hunt PW , Sinclair E , Rodriguez B , Shive C , Clagett B , Funderburg N , Robinson J , Huang Y , Epling L , Martin JN , et al. Gut epithelial barrier dysfunction and innate immune activation predict mortality in treated HIV infection. J Infect Dis. 2014;210(8):1228–1238. doi: 10.1093/infdis/jiu238. 24755434 PMC4192038

[28] Alonso A , Barnes AE , Guest JL , Shah A , Shao IY , Marconi V . HIV infection and incidence of cardiovascular diseases: an analysis of a large healthcare database. J Am Heart Assoc. 2019;8(14):e012241. doi: 10.1161/JAHA.119.012241. 31266386 PMC6662120

[29] Masiá M , Padilla S , García JA , García-Abellán J , Fernández M , Bernardino I , Montero M , Peraire J , Pernas B , Gutiérrez F , et al. Evolving understanding of cardiovascular, cerebrovascular and peripheral arterial disease in people living with HIV and role of novel biomarkers. A study of the spanish CoRIS cohort, 2004-2015. PLoS One. 2019 Apr 26;14(4):e0215507. doi: 10.1371/journal.pone.0215507. 31026289 PMC6485642

[30] Masiá M , García JA , García-Abellán J , Padilla S , Fernández-González M , Agulló V , Gosalbes MJ , Ruíz-Pérez S , Mascarell P , Botella A , et al. Distinct gut microbiota signatures associated with progression of atherosclerosis in people living with human immunodeficiency virus. J Infect Dis. 2025;231(6):1371–1381. doi: 10.1093/infdis/jiae243. 38743815

[31] Toya T , Corban MT , Marrietta E , Horwath IE , Lerman LO , Murray JA , Pizzi C . Coronary artery disease is associated with an altered gut microbiome composition. PLoS One. 2020;15(1):e0227147. doi: 10.1371/journal.pone.0227147. 31995569 PMC6988937

[32] Emoto T , Yamashita T , Sasaki N , Hirota Y , Hayashi T , So A , Kasahara K , Yodoi K , Matsumoto T , Mizoguchi T , et al. Analysis of gut microbiota in coronary artery disease patients: a possible link between gut microbiota and coronary artery disease. J Atheroscler Thromb. 2016;23(8):908–921. doi: 10.5551/jat.32672. 26947598 PMC7399299

[33] Sayols-Baixeras S , Dekkers KF , Baldanzi G , Jönsson D , Hammar U , Lin YT , Ahmad S , Nguyen D , Varotsis G , Pita S , et al. Streptococcus species abundance in the gut is linked to subclinical coronary atherosclerosis in 8973 participants from the SCAPIS cohort. Circulation. 2023;148:459–472. doi: 10.1161/CIRCULATIONAHA.123.063914. 37435755 PMC10399955

[34] Liu C , Sun Z , Shali S , Mei Z , Chang S , Mo H , Xu L , Pu Y , Guan H , Chen G , et al. The gut microbiome and microbial metabolites in acute myocardial infarction. J Genet Genomics. 2022;49:569–578. doi: 10.1016/j.jgg.2021.12.007. 34974193

[35] Hodgkinson K , El Abbar F , Dobranowski P , Manoogian J , Butcher J , Figeys D , Mack D , Stintzi A . Butyrate's role in human health and the current progress towards its clinical application to treat gastrointestinal disease. Clin Nutr. 2023;42(2):61–75. doi: 10.1016/j.clnu.2022.10.024. 36502573

[36] Nageswaran V , Carreras A , Reinshagen L , Beck KR , Steinfeldt J , Henricsson M , Ramezani Rad P , Peters L , Strässler ET , Lim J , et al. Gut microbial metabolite imidazole propionate impairs endothelial cell function and promotes the development of atherosclerosis. Arterioscler Thromb Vasc Biol. 2025;45(5):823–839. doi: 10.1161/ATVBAHA.124.322346. 40143816 PMC12017598

[37] (a) Cai YY , Huang FQ , Lao X , Lu Y , Gao X , Alolga RN , Yin K , Zhou X , Wang Y , Liu B , et al. Integrated metagenomics identifies a crucial role for trimethylamine-producing lachnoclostridium in promoting atherosclerosis. NPJ Biofilms Microbiomes. 2022;8(1):11. doi: 10.1038/s41522-022-00273-4. 35273169 PMC8913745

[38] Teng D , Jia W , Wang W , Liao L , Xu B , Gong L , Dong H , Zhong L , Yang J . Causality of the gut microbiome and atherosclerosis-related lipids: a bidirectional mendelian randomization study. BMC Cardiovasc Disord. 2024;24(1):138. doi: 10.1186/s12872-024-03804-3. 38431594 PMC10909291

[39] Li Y , Chen Y , Li Z , Li Y , Chen Y , Tang L . Gut microbiome and atherosclerosis: a mendelian randomization study. Rev Cardiovasc Med. 2024;25(2):41. doi: 10.31083/j.rcm2502041. 39077366 PMC11263158

[40] Liu H , Tian R , Wang H , Feng S , Li H , Xiao Y , Luan X , Zhang Z , Shi N , Niu H . Gut microbiota from coronary artery disease patients contributes to vascular dysfunction in mice by regulating bile acid metabolism and immune activation. J Transl Med. 2020;18(1):382. doi: 10.1186/s12967-020-02539-x. 33036625 PMC7547479

[41] Wang Z , Roberts AB , Buffa JA , Levison BS , Zhu W , Org E , Gu X , Huang Y , Zamanian-Daryoush M , Culley MK , et al. Non-lethal inhibition of gut microbial trimethylamine production for the treatment of atherosclerosis. Cell. 2015;163(7):1585–1595. doi: 10.1016/j.cell.2015.11.055. 26687352 PMC4871610

[42] Tang WH , Wang Z , Levison BS , Koeth RA , Britt EB , Fu X , Wu Y , Hazen SL . Intestinal microbial metabolism of phosphatidylcholine and cardiovascular risk. NEJM. 2013;368(17):1575–1584. doi: 10.1056/NEJMoa1109400. 23614584 PMC3701945

[43] Randrianarisoa E , Lehn-Stefan A , Wang X , Hoene M , Peter A , Heinzmann SS , Zhao X , Königsrainer I , Balletshofer B , Machann J , et al. Relationship of serum trimethylamine N-Oxide (TMAO) levels with early atherosclerosis in humans. Sci Rep. 2016;6:26745. doi: 10.1038/srep26745. 27228955 PMC4882652

[44] Senthong V , Li XS , Hudec T , Coughlin J , Wu Y , Levison B , Wang Z , Hazen SL , Tang WW . Plasma trimethylamine N-Oxide, a gut microbe-generated phosphatidylcholine metabolite, is associated with atherosclerotic burden. J Am Coll Cardiol. 2016;67(22):2620–2628. doi: 10.1016/j.jacc.2016.03.546. 27256833 PMC4893167

[45] Ortega-Madueño I , Modrego J , Gómez-Gordo R , Ortega-Hernández A , Pérez de Isla L , Muñoz JC , Nieto ML , Gómez-Garre D . Relationship between the coronary artery calcium quantification and gut microbiota composition in subjects without previous cardiovascular disease: a pilot study. Clin Investig Arterioscler. 2022;34(4):205–215. doi: 10.1016/j.arteri.2021.11.008 English, Spanish. 35125248

[46] Menni C , Mangino M , Cecelja M , Psatha M , Brosnan MJ , Trimmer J , Mohney RP , Chowienczyk P , Padmanabhan S , Spector TD , et al. Metabolomic study of carotid-femoral pulse-wave velocity in women. J Hypertens. 2015;33(4):791–796. doi: 10.1097/HJH.0000000000000467. 25490711 PMC4354457

[47] Liu Y , Liu S , Zhao Z , Song X , Qu H , Liu H . Phenylacetylglutamine is associated with the degree of coronary atherosclerotic severity assessed by coronary computed tomographic angiography in patients with suspected coronary artery disease. Atherosclerosis. 2021;333:75–82. doi: 10.1016/j.atherosclerosis.2021.08.029. 34438323

[48] Bartolomaeus H , Balogh A , Yakoub M , Homann S , Markó L , Höges S , Tsvetkov D , Krannich A , Wundersitz S , Avery EG , et al. Short-chain fatty acid propionate protects from hypertensive cardiovascular damage. Circulation. 2019;139(11):1407–1421. doi: 10.1161/CIRCULATIONAHA.118.036652. 30586752 PMC6416008

[49] Brown AJ , Goldsworthy SM , Barnes AA , Eilert MM , Tcheang L , Daniels D , Muir AI , Wigglesworth MJ , Kinghorn I , Fraser NJ , et al. The orphan G protein-coupled receptors GPR41 and GPR43 are activated by propionate and other short chain carboxylic acids. J Biol Chem. 2003;278(13):11312–11319. doi: 10.1074/jbc.M211609200. 12496283

[50] Pluznick JL , Protzko RJ , Gevorgyan H , Peterlin Z , Sipos A , Han J , Brunet I , Wan L , Rey F , Wang T , et al. Olfactory receptor responding to gut microbiota-derived signals plays a role in renin secretion and blood pressure regulation. Proc Natl Acad Sci U S A. 2013;110(11):4410–4415. doi: 10.1073/pnas.1215927110. 23401498 PMC3600440

[51] Hylemon PB , Zhou H , Pandak WM , Ren S , Gil G , Dent P . Bile acids as regulatory molecules. J Lipid Res. 2009;50(8):1509–1520. doi: 10.1194/jlr.R900007-JLR200. 19346331 PMC2724047

[52] Kida T , Tsubosaka Y , Hori M , Ozaki H , Murata T . Bile acid receptor TGR5 agonism induces NO production and reduces monocyte adhesion in vascular endothelial cells. Arterioscler Thromb Vasc Biol. 2013;33(7):1663–1669. doi: 10.1161/ATVBAHA.113.301565. 23619297

[53] (a) Imhann F , Vich Vila A , Bonder MJ , Lopez Manosalva AG , Koonen DPY , Fu J , Wijmenga C , Zhernakova A , Weersma RK . The influence of proton pump inhibitors and other commonly used medication on the gut microbiota. Gut Microbes. 2017;8(4):351–358. doi: 10.1080/19490976.2017.1284732. 28118083 PMC5570416

[54] Vich Vila A , Collij V , Sanna S , Sinha T , Imhann F , Bourgonje AR , Mujagic Z , Jonkers DMAE , Masclee AAM , Fu J , et al. Impact of commonly used drugs on the composition and metabolic function of the gut microbiota. Nat Commun. 2020;11(1):362. doi: 10.1038/s41467-019-14177-z. 31953381 PMC6969170

[55] Li X , Li Q , Wang L , Ding H , Wang Y , Liu Y , Gong T . The interaction between oral microbiota and gut microbiota in atherosclerosis. Front Cardiovasc Med. 2024 Jun 12;11:1406220. doi: 10.3389/fcvm.2024.1406220. 38932989 PMC11199871

[56] Cani PD , Amar J , Iglesias MA , Poggi M , Knauf C , Bastelica D , Neyrinck AM , Fava F , Tuohy KM , Chabo C , et al. Metabolic endotoxemia initiates obesity and insulin resistance. Diabetes. 2007;56(7):1761–1772. doi: 10.2337/db06-1491. 17456850

[57] Clarke TB , Davis KM , Lysenko ES , Zhou AY , Yu Y , Weiser JN . Recognition of peptidoglycan from the microbiota by Nod1 enhances systemic innate immunity. Nat Med. 2010;16(2):228–231. doi: 10.1038/nm.2087. 20081863 PMC4497535

[58] Akira S , Uematsu S , Takeuchi O . Pathogen recognition and innate immunity. Cell. 2006;124(4):783–801. doi: 10.1016/j.cell.2006.02.015. 16497588

[59] Erridge C . The capacity of foodstuffs to induce innate immune activation of human monocytes in vitro is dependent on food content of stimulants of toll-like receptors 2 and 4. Br J Nutr. 2011;105(1):15–23. doi: 10.1017/S0007114510003004. 20849668

[60] Lepper PM , Kleber ME , Grammer TB , Hoffmann K , Dietz S , Winkelmann BR , Boehm BO , März W . Lipopolysaccharide-binding protein (LBP) is associated with total and cardiovascular mortality in individuals with or without stable coronary artery disease-results from the ludwigshafen risk and cardiovascular health study (LURIC). Atherosclerosis. 2011;219(1):291–297. doi: 10.1016/j.atherosclerosis.2011.06.001. 21722903

[61] Lassenius MI , Pietiläinen KH , Kaartinen K , Pussinen PJ , Syrjänen J , Forsblom C , Pörsti I , Rissanen A , Kaprio J , Mustonen J , et al. FinnDiane Study Group Bacterial endotoxin activity in human serum is associated with dyslipidemia, insulin resistance, obesity, and chronic inflammation. Diabetes Care. 2011;34(8):1809–1815. doi: 10.2337/dc10-2197. 21636801 PMC3142060

[62] Wang H , Sun X , Lu Q , Zemskov EA , Yegambaram M , Wu X , Tang H , Black SM . The mitochondrial redistribution of eNOS is involved in lipopolysaccharide induced inflammasome activation during acute lung injury. Redox Biol. 2021;41:101878. doi: 10.1016/j.redox.2021.101878. 33578126 PMC7879038

[63] Fang Y , Meng H , Wang J . Mechanisms of LPS-induced toxicity in endothelial cells and the protective role of geniposidic acid. Food Chem Toxicol. 2025;201:115488. doi: 10.1016/j.fct.2025.115488. 40288513

[64] (a) Thevaranjan N , Puchta A , Schulz C , Naidoo A , Szamosi JC , Verschoor CP , Loukov D , Schenck LP , Jury J , Foley KP , et al. Age-associated microbial dysbiosis promotes intestinal permeability, systemic inflammation, and macrophage dysfunction. Cell Host Microbe. 2017;21(4):455–466.e4. doi: 10.1016/j.chom.2017.03.002. 28407483 PMC5392495

[65] Mishra SP , Jain S , Wang B , Wang S , Miller BC , Lee JY , Borlongan CV , Jiang L , Pollak J , Taraphder S , et al. Abnormalities in microbiota/butyrate/FFAR3 signaling in aging gut impair brain function. JCI Insight. 2024;9(3):e168443. doi: 10.1172/jci.insight.168443. 38329121 PMC10967378

[66] Warnatsch A , Ioannou M , Wang Q , Papayannopoulos V . Inflammation. Neutrophil extracellular traps license macrophages for cytokine production in atherosclerosis. Science. 2015;349(6245):316–320. doi: 10.1126/science.aaa8064. 26185250 PMC4854322

[67] Josefs T , Barrett TJ , Brown EJ , Quezada A , Wu X , Voisin M , Amengual J , Fisher EA . Neutrophil extracellular traps promote macrophage inflammation and impair atherosclerosis resolution in diabetic mice. JCI Insight. 2020;5(7):e134796. doi: 10.1172/jci.insight.134796. 32191637 PMC7205252

[68] Taleb S , Tedgui A , Mallat Z . IL-17 and Th17 cells in atherosclerosis: subtle and contextual roles. Arterioscler Thromb Vasc Biol. 2015;35(2):258–264. doi: 10.1161/ATVBAHA.114.303567. 25234818

[69] Erbel C , Akhavanpoor M , Okuyucu D , Wangler S , Dietz A , Zhao L , Stellos K , Little KM , Lasitschka F , Doesch A , et al. IL-17A influences essential functions of the monocyte/macrophage lineage and is involved in advanced murine and human atherosclerosis. J Immunol. 2014;193(9):4344–4355. doi: 10.4049/jimmunol.1400181. 25261478 PMC4201987

[70] Kyaw T , Tay C , Khan A , Dumouchel V , Cao A , To K , Kehry M , Dunn R , Agrotis A , Tipping P , et al. Conventional B2 B cell depletion ameliorates whereas its adoptive transfer aggravates atherosclerosis. J Immunol. 2010;185(7):4410–4419. doi: 10.4049/jimmunol.1000033. 20817865

[71] Chen K , Magri G , Grasset EK , Cerutti A . Rethinking mucosal antibody responses: IgM, IgG and IgD join IgA. Nat Rev Immunol. 2020;20(7):427–441. doi: 10.1038/s41577-019-0261-1. 32015473 PMC10262260

[72] Winter SE , Winter MG , Xavier MN , Thiennimitr P , Poon V , Keestra AM , Laughlin RC , Gomez G , Wu J , Lawhon SD , et al. Host-derived nitrate boosts growth of E. Coli in the inflamed gut. Science. 2013;339(6120):708–711. doi: 10.1126/science.1232467. 23393266 PMC4004111

[73] Byndloss MX , Olsan EE , Rivera-Chávez F , Tiffany CR , Cevallos SA , Lokken KL , Torres TP , Faber F , Gao Y , Litvak Y , et al. Microbiota-activated PPAR-γ signaling inhibits dysbiotic enterobacteriaceae expansion. Science. 2017;357(6351):570–575. doi: 10.1126/science.aam9949. 28798125 PMC5642957

[74] Zhu W , Gregory JC , Org E , Buffa JA , Gupta N , Wang Z , Li L , Fu X , Wu Y , Mehrabian M , et al. Gut microbial metabolite TMAO enhances platelet hyperreactivity and thrombosis risk. Cell. 2016;165(1):111–124. doi: 10.1016/j.cell.2016.02.011. 26972052 PMC4862743

[75] Brunt VE , Gioscia-Ryan RA , Casso AG , VanDongen NS , Ziemba BP , Sapinsley ZJ , Richey JJ , Zigler MC , Neilson AP , Davy KP , et al. Trimethylamine-N-Oxide promotes age-related vascular oxidative stress and endothelial dysfunction in mice and healthy humans. Hypertension. 2020;76(1):101–112. doi: 10.1161/HYPERTENSIONAHA.120.14759. 32520619 PMC7295014

[76] Warrier M , Shih DM , Burrows AC , Ferguson D , Gromovsky AD , Brown AL , Marshall S , McDaniel A , Schugar RC , Wang Z , et al. The TMAO-Generating enzyme flavin monooxygenase 3 is a central regulator of cholesterol balance. Cell Rep. 2015;10(3):326–338. doi: 10.1016/j.celrep.2014.12.036. 25600868 PMC4501903

[77] Molinaro A , Nemet I , Bel Lassen P , Chakaroun R , Nielsen T , Aron-Wisnewsky J , Bergh P , Li L , Henricsson M , Køber L , et al. MetaCardis consortium. Microbially produced imidazole propionate is associated with heart failure and mortality. JACC Heart Fail. 2023;11(7):810–821. doi: 10.1016/j.jchf.2023.03.008. 37115134 PMC12512386

[78] Wenzl FA , Wang P , Kahles F , Beck KR , Giannitsis E , Obeid S , Bruno F , Li XS , Nanchen D , Liberale L , et al. Gut microbiota-derived imidazole propionate predicts cardiometabolic risk in patients with coronary artery disease. Eur Heart J. 2026;47(31):4259–4273. doi: 10.1093/eurheartj/ehaf661. 40884168 PMC13472685

[79] Tuomainen M , Lindström J , Lehtonen M , Auriola S , Pihlajamäki J , Peltonen M , Tuomilehto J , Uusitupa M , Mello VD , Hanhineva K . Associations of serum indolepropionic acid, a gut microbiota metabolite, with type 2 diabetes and low-grade inflammation in high-risk individuals. Nutr & Diabetes. 2018;8:35. doi: 10.1038/s41387-018-0046-9. PMC5968030 29795366

[80] Xue H , Chen X , Yu C , Deng Y , Zhang Y , Chen S , Yang Y , Ling W . Gut microbially produced Indole-3-Propionic acid inhibits atherosclerosis by promoting reverse cholesterol transport and its deficiency is causally related to atherosclerotic cardiovascular disease. Circ Res. 2022;131(5):404–420. doi: 10.1161/CIRCRESAHA.122.321253. 35893593

[81] Luo K , Wang Z , Peters BA , Hanna DB , Wang T , Sollecito CC , Grassi E , Wiek F , St. Peter L , Usyk M , et al. Tryptophan metabolism, gut microbiota, and carotid artery plaque in women with and without HIV infection. AIDS. 2024;38(2):223–233. doi: 10.1097/QAD.0000000000003596. 37199567 PMC10640661

[82] Venkatesh M , Mukherjee S , Wang H , Li H , Sun K , Benechet AP , Qiu Z , Maher L , Redinbo MR , Phillips RS , et al. Symbiotic bacterial metabolites regulate gastrointestinal barrier function via the xenobiotic sensor PXR and toll-like receptor 4. Immunity. 2014;41(2):296–310. doi: 10.1016/j.immuni.2014.06.014. 25065623 PMC4142105

[83] Ridlon JM , Kang DJ , Hylemon PB , Bajaj JS . Bile acids and the gut microbiome. Curr Opin Gastroenterol. 2014 May;30(3):332–338. doi: 10.1097/MOG.0000000000000057. 24625896 PMC4215539

[84] Hanniman EA , Lambert G , McCarthy TC , Sinal CJ . Loss of functional farnesoid X receptor increases atherosclerotic lesions in apolipoprotein E-deficient mice. J Lipid Res. 2005;46(12):2595–2604. doi: 10.1194/jlr.M500390-JLR200. 16186601

[85] Shi H , Wu M , Wu X , Liu Z , Jiang S , Li G , Yang Y , Fu Y , Wang Q , Zhang G , et al. Multi-omics integration reveals functional signatures of gut microbiome in atherosclerosis. Gut Microbes. 2025;17(1):2542384. doi: 10.1080/19490976.2025.2542384. 40785047 PMC12341049

[86] Carreras-Torres R , Galván-Femenía I , Farré X , Cortés B , Díez-Obrero V , Carreras A , Moratalla-Navarro F , Iraola-Guzmán S , Blay N , Obón-Santacana M , et al. Multiomic integration analysis identifies atherogenic metabolites mediating between novel immune genes and cardiovascular risk. Genome Med. 2024;16(1):122. doi: 10.1186/s13073-024-01397-2. 39449064 PMC11515386

[87] Nambi V , Chambless L , Folsom AR , He M , Hu Y , Mosley T , Volcik K , Boerwinkle E , Ballantyne CM . Carotid intima-media thickness and presence or absence of plaque improves prediction of coronary heart disease risk: the ARIC (Atherosclerosis Risk In Communities) study. J Am Coll Cardiol. 2010;55(15):1600–1607. doi: 10.1016/j.jacc.2009.11.075. 20378078 PMC2862308

[88] (a) Mehta A , Pandey A , Ayers CR , Khera A , Sperling LS , Szklo MS , Gottesman RF , Budoff MJ , Blaha MJ , Blumenthal RS , et al. Predictive value of coronary artery calcium score categories for coronary events versus strokes: impact of sex and race: MESA and DHS. Circ Cardiovasc Imaging. 2020;13(8), e010153. doi: 10.1161/CIRCIMAGING.119.010153. 32806939 PMC7440773

[89] Srinivasa S , Fitch KV , Lo J , Kadar H , Knight R , Wong K , Abbara S , Gauguier D , Capeau J , Boccara F , et al. Plaque burden in HIV-infected patients is associated with serum intestinal microbiota-generated trimethylamine. AIDS. 2015;29(4):443–452. doi: 10.1097/QAD.0000000000000565. 25565500 PMC4410017

[90] Shan Z , Clish CB , Hua S , Scott JM , Hanna DB , Burk RD , Haberlen SA , Shah SJ , Margolick JB , Sears CL , et al. Gut microbial-related choline metabolite trimethylamine-N-oxide is associated with progression of carotid artery atherosclerosis in HIV infection. J Infect Dis. 2018;218(9):1474–1479. doi: 10.1093/infdis/jiy356. 29912352 PMC6151074

[91] Missailidis C , Neogi U , Stenvinkel P , Trøseid M , Nowak P , Bergman P . The microbial metabolite trimethylamine-N-oxide in association with inflammation and microbial dysregulation in three HIV cohorts at various disease stages. AIDS. 2018;32(12):1589–1598. doi: 10.1097/QAD.0000000000001813. 29620717

[92] García-Abellán J , García JA , Galdámez R , Gosalbes MJ , Fernández-González M , Padilla S , Mascarell P , Telenti G , Gutiérrez F , Masiá M . Gut microbiota and weight gain in people with HIV on integrase inhibitors: a five-year longitudinal study. J Antimicrob Chemother. 2025;80(8):2145–2157. doi: 10.1093/jac/dkaf182. 40470774

[93] Trøseid M , Molinaro A , Gelpi M , Vestad B , Kofoed KF , Fuchs A , Køber L , Holm K , Benfield T , Ueland PM , et al. Gut microbiota alterations and circulating imidazole propionate levels are associated with obstructive coronary artery disease in people with HIV. J Infect Dis. 2024;229(3):898–907. doi: 10.1093/infdis/jiad604. 38195204 PMC10938217

[94] Stubbs JR , House JA , Ocque AJ , Zhang S , Johnson C , Kimber C , Schmidt K , Gupta A , Wetmore JB , Nolin TD , et al. Serum Trimethylamine-N-Oxide is elevated in CKD and correlates with coronary atherosclerosis burden. J Am Soc Nephrol. 2016;27(1):305–313. doi: 10.1681/ASN.2014111063. 26229137 PMC4696571

[95] Poesen R , Claes K , Evenepoel P , de Loor H , Augustijns P , Kuypers D , Meijers B . Microbiota-derived phenylacetylglutamine associates with overall mortality and cardiovascular disease in patients with CKD. J Am Soc Nephrol. 2016;27(11):3479–3487. doi: 10.1681/ASN.2015121302. 27230658 PMC5084895

[96] Stremmel W , Schmidt KV , Schuhmann V , Kratzer F , Garbade SF , Langhans CD , Fricker G , Okun JG , Nerurkar PV . Blood Trimethylamine-N-Oxide originates from microbiota mediated breakdown of phosphatidylcholine and absorption from small intestine. PLoS One. 2017;12(1):e0170742. doi: 10.1371/journal.pone.0170742. 28129384 PMC5271338

[97] Chen CY , Leu HB , Wang SC , Tsai SH , Chou RH , Lu YW , Kuo C , Huang P , Lin S . Inhibition of trimethylamine N-Oxide attenuates neointimal formation through reduction of inflammasome and oxidative stress in a mouse model of carotid artery ligation. Antioxid Redox Signal. 2023;38(1-3):215–233. doi: 10.1089/ars.2021.0115. 35713239

[98] Zouiouich S , Loftfield E , Huybrechts I , Viallon V , Louca P , Vogtmann E , Wells PM , Steves CJ , Herzig K , Menni C , et al. Markers of metabolic health and gut microbiome diversity: findings from two population-based cohort studies. Diabetologia. 2021;64(8):1749–1759. doi: 10.1007/s00125-021-05464-w. 34110438 PMC8245388

[99] Vadaq N , Zhang Y , Vos WA , Groenendijk AL , Blaauw MJ , van Eekeren LE , Jacobs-Cleophas M , van de Wijer L , dos Santos JC , Gasem MH , et al. High-throughput proteomic analysis reveals systemic dysregulation in virally suppressed people living with HIV. JCI Insight. 2023;8(11):e166166. doi: 10.1172/jci.insight.166166. 37079385 PMC10393229

[100] Folkersen L , Gustafsson S , Wang Q , Hansen DH , Hedman [ , Schork A , Page K , Zhernakova DV , Wu Y , Peters J , et al. Genomic and drug target evaluation of 90 cardiovascular proteins in 30,931 individuals. Nat Metab. 2020;2(10):1135–1148. doi: 10.1038/s42255-020-00287-2. 33067605 PMC7611474

[101] Royer P , Björnson E , Adiels M , Álvez MB , Fagerberg L , Bäckhed F , Uhlén M , Gummesson A , Bergström G . Plasma proteomics for prediction of subclinical coronary artery calcifications in primary prevention. Am Heart J. 2024;271:55–67. doi: 10.1016/j.ahj.2024.01.011. 38325523

[102] Zierer J , Jackson MA , Kastenmüller G , Mangino M , Long T , Telenti A , Mohney RP , Small KS , Bell JT , Steves CJ , et al. The fecal metabolome as a functional readout of the gut microbiome. Nat Genet. 2018;50(6):790–795. doi: 10.1038/s41588-018-0135-7. 29808030 PMC6104805

[103] Ruschitzka F , Vidal-Puig A , Saeedi Saravi SS . Gut microbial metabolite connections to cardiovascular disease call for gutsy therapeutic approaches. J Clin Invest. 2025;135(23):e201468. doi: 10.1172/JCI201468. 41321308 PMC12646642

[104] An K , Jia Y , Xie B , Gao J , Chen Y , Yuan W , Zhong J , Su P , Liu X . Alterations in the gut mycobiome with coronary artery disease severity. EBioMedicine. 2024;103:105137. doi: 10.1016/j.ebiom.2024.105137. 38703606 PMC11087906

[105] Triant VA , Perez J , Regan S , Massaro JM , Meigs JB , Grinspoon SK , D'Agostino RB, Sr . Cardiovascular risk prediction functions underestimate risk in HIV infection. Circulation. 2018;137(21):2203–2214. doi: 10.1161/CIRCULATIONAHA.117.028975. 29444987 PMC6157923

[106] Duprez DA , Neuhaus J , Kuller LH , Tracy R , Belloso W , De Wit S , Drummond F , Lane HC , Ledergerber B , Lundgren J , et al. INSIGHT SMART study group. Inflammation, coagulation and cardiovascular disease in HIV-infected individuals. PLoS One. 2012;7(9):e44454. doi: 10.1371/journal.pone.0044454. 22970224 PMC3438173

[107] Safo SE , Haine L , Baker J , Reilly C , Duprez D , Neaton JD , Jain MK , Arenas‐Pinto A , Polizzotto M , Staub T . ESPRIT, INSIGHT FIRST, SMART and START study groups. Derivation of a protein risk score for cardiovascular disease among a multiracial and multiethnic HIV+ cohort. J Am Heart Assoc. 2023;12(13):e027273. doi: 10.1161/JAHA.122.027273. 37345752 PMC10356060

[108] (a) Vujkovic-Cvijin I , Dunham RM , Iwai S , Maher MC , Albright RG , Broadhurst MJ , Hernandez RD , Lederman MM , Huang Y , Somsouk M , et al. Dysbiosis of the gut microbiota is associated with HIV disease progression and tryptophan catabolism. Sci Transl Med. 2013;5(193):193ra91. doi: 10.1126/scitranslmed.3006438. PMC4094294 23843452

[109] Nowak P , Troseid M , Avershina E , Barqasho B , Neogi U , Holm K , Hov JR , Noyan K , Vesterbacka J , Svärd J , et al. Gut microbiota diversity predicts immune status in HIV-1 infection. AIDS. 2015;29(18):2409–2418. doi: 10.1097/QAD.0000000000000869. 26355675

[110] Vázquez-Castellanos JF , Serrano-Villar S , Latorre A , Artacho A , Ferrús ML , Madrid N , Vallejo A , Sainz T , Martínez-Botas J , Ferrando-Martínez S , et al. Altered metabolism of gut microbiota contributes to chronic immune activation in HIV-infected individuals. Mucosal Immunol. 2015;8(4):760–772. doi: 10.1038/mi.2014.107. 25407519

[111] Serrano-Villar S , Rojo D , Martínez-Martínez M , Deusch S , Vázquez-Castellanos JF , Bargiela R , Sainz T , Vera M , Moreno S , Estrada V , et al. Gut bacteria metabolism impacts immune recovery in HIV-infected individuals. EBioMedicine. 2016 Jun;8:203–216. doi: 10.1016/j.ebiom.2016.04.033. 27428431 PMC4919658

[112] Miller PE , Haberlen SA , Brown TT , Margolick JB , DiDonato JA , Hazen SL , Witt MD , Kingsley LA , Palella FJ , Budoff M , et al. Brief report: intestinal microbiota-produced trimethylamine-N-oxide and its association with coronary stenosis and HIV serostatus. J Acquir Immune Defic Syndr. 2016;72(1):114–118. doi: 10.1097/QAI.0000000000000937. 26825179 PMC4836953

[113] Haissman JM , Haugaard AK , Ostrowski SR , Berge RK , Hov JR , Trøseid M , Nielsen SD . Microbiota-dependent metabolite and cardiovascular disease marker trimethylamine-N-oxide (TMAO) is associated with monocyte activation but not platelet function in untreated HIV infection. BMC Infect Dis. 2017;17(1):445. doi: 10.1186/s12879-017-2547-x. 28645263 PMC5481962

[114] Raju SC , Molinaro A , Awoyemi A , Jørgensen SF , Braadland PR , Nendl A , Seljeflot I , Ueland PM , McCann A , Aukrust P , et al. Microbial-derived imidazole propionate links the heart failure-associated microbiome alterations to disease severity. Genome Med. 2024;16(1):27. doi: 10.1186/s13073-024-01296-6. 38331891 PMC10854170

[115] Park H , Cheon J , Kim H , Kim J , Kim J , Shin JY , Ryu G , Chung IY , Zhang Z , Wu H , et al. Gut microbial production of imidazole propionate drives Parkinson's pathologies. Nat Commun. 2025;16(1):8216. doi: 10.1038/s41467-025-63473-4. 40913049 PMC12413466

[116] Skye SM , Zhu W , Romano KA , Guo CJ , Wang Z , Jia X , Kirsop J , Haag B , Lang JM , DiDonato JA , et al. Microbial transplantation with human gut commensals containing CutC is sufficient to transmit enhanced platelet reactivity and thrombosis potential. Circ Res. 2018;123(10):1164–1176. doi: 10.1161/CIRCRESAHA.118.313142. 30359185 PMC6223262

[117] Wilkinson MD , Dumontier M , Aalbersberg IJ , Appleton G , Axton M , Baak A , Blomberg N , Boiten J , da Silva Santos LB , Bourne PE , et al. The FAIR guiding principles for scientific data management and stewardship. Sci Data. 2016;3:160018. doi: 10.1038/sdata.2016.18. 26978244 PMC4792175

[118] Ahrens AP , Culpepper T , Saldivar B , Anton S , Stoll S , Handberg EM , Xu K , Pepine C , Triplett EW , Aggarwal M . A six-day, lifestyle-based immersion program mitigates cardiovascular risk factors and induces shifts in gut microbiota, specifically lachnospiraceae, ruminococcaceae, faecalibacterium prausnitzii: a pilot study. Nutrients. 2021;13(10):3459. doi: 10.3390/nu13103459. 34684459 PMC8539164

[119] Falony G , Joossens M , Vieira-Silva S , Wang J , Darzi Y , Faust K , Kurilshikov A , Bonder MJ , Valles-Colomer M , Vandeputte D , et al. Population-level analysis of gut microbiome variation. Science. 2016;352(6285):560–564. doi: 10.1126/science.aad3503. 27126039

[120] Malik M , Suboc TM , Tyagi S , Salzman N , Wang J , Ying R , Tanner MJ , Kakarla M , Baker JE , Widlansky ME . Lactobacillus plantarum 299v supplementation improves vascular endothelial function and reduces inflammatory biomarkers in men with stable coronary artery disease. Circ Res. 2018;123(9):1091–1102. doi: 10.1161/CIRCRESAHA.118.313565. 30355158 PMC6205737

[121] Marques FZ , Nelson E , Chu PY , Horlock D , Fiedler A , Ziemann M , Tan JK , Kuruppu S , Rajapakse NW , El-Osta A , et al. High-fiber diet and acetate supplementation change the gut microbiota and prevent the development of hypertension and heart failure in hypertensive mice. Circulation. 2017;135(10):964–977. doi: 10.1161/CIRCULATIONAHA.116.024545. 27927713

[122] Vrieze A , Van Nood E , Holleman F , Salojärvi J , Kootte RS , Bartelsman JF , Dallinga–Thie GM , Ackermans MT , Serlie MJ , Oozeer R , et al. Transfer of intestinal microbiota from lean donors increases insulin sensitivity in individuals with metabolic syndrome. Gastroenterology. 2012;143(4):913–6.e7. doi: 10.1053/j.gastro.2012.06.031. 22728514

[123] Depommier C , Everard A , Druart C , Plovier H , Van Hul M , Vieira-Silva S , Falony G , Raes J , Maiter D , Delzenne NM , et al. Supplementation with akkermansia muciniphila in overweight and obese human volunteers: a proof-of-concept exploratory study. Nat Med. 2019;25(7):1096–1103. doi: 10.1038/s41591-019-0495-2. 31263284 PMC6699990

[124] Sokol H , Pigneur B , Watterlot L , Lakhdari O , Bermúdez-Humarán LG , Gratadoux JJ , Blugeon S , Bridonneau C , Furet J , Corthier G , et al. Faecalibacterium prausnitzii is an anti-inflammatory commensal bacterium identified by gut microbiota analysis of crohn disease patients. Proc Natl Acad Sci U S A. 2008;105(43):16731–16736. doi: 10.1073/pnas.0804812105. 18936492 PMC2575488

[125] Sinha R , Abnet CC , White O , Knight R , Huttenhower C . The microbiome quality control project: baseline study design and future directions. Genome Biol. 2015;16:276. doi: 10.1186/s13059-015-0841-8. 26653756 PMC4674991

[126] Sinha R , Abu-Ali G , Vogtmann E , Fodor AA , Ren B , Amir A , Schwager E , Crabtree J , Ma S , Abnet CC , et al. Assessment of variation in microbial community amplicon sequencing by the microbiome quality control (MBQC) project consortium. Nat Biotechnol. 2017;35(11):1077–1086. doi: 10.1038/nbt.3981. 28967885 PMC5839636

[127] Mirzayi C , Renson A , Zohra F , Elsafoury S , Kasselman L , van de Wijgert J , Furlanello C , Sansone S , Geistlinger L , Eckenrode K , et al. Reporting guidelines for human microbiome research: the STORMS checklist. Nat Med. 2021;27(11):1885–1892. doi: 10.1038/s41591-021-01552-x. 34789871 PMC9105086

